# Supplementary figures and images for: Evolutionary trade-off and mutational bias could favor transcriptional over translational divergence within paralog pairs
Source: PLoS Genet. 2023 May 26;19(5):e1010756. doi: 10.1371/journal.pgen.1010756 (PMC10275480; doi:10.1371/journal.pgen.1010756)

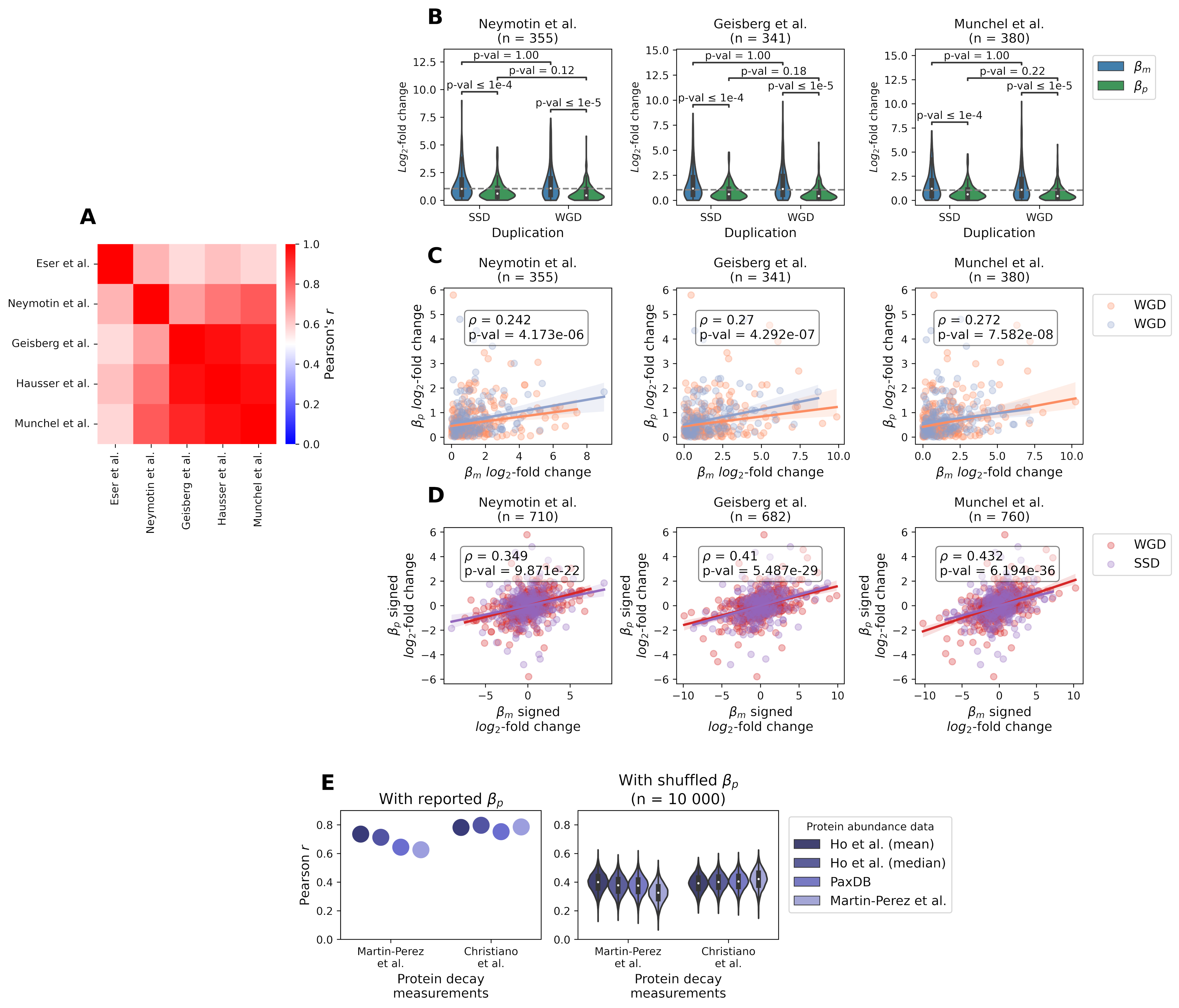

Supplement: S1 Fig — (A) Correlations between the different sets of transcription rates βm used. The rates originally reported by [25] are identified as “Hausser et al.”, while the four others are βm recomputed using the data from [25] and the corresponding set of gene-specific experimental measurements of transcript decay [26–29]. (B) Distributions of relative divergence in transcription and in translation for S. cerevisiae paralog pairs by duplication mechanism. Transcription rates have been recalculated using the corresponding set of mRNA decay measurements, while translation rates are the same as in Fig 1. P-values from Mann-Whitney-Wilcoxon two-sided tests are shown. (C) Correlation (Spearman’s ρ) between the magnitudes of relative divergence in transcription and translation rates across all paralog pairs, using βm rates recalculated when accounting for variations in transcript decay rate. (D) Correlation (Spearman’s ρ) between the signed relative divergences in transcription and in translation for all gene pairs, when βm is calculated using the corresponding mRNA decay measurements. Each correlation was computed on a duplicated dataset, obtained by calculating the signed log2-fold changes in the two possible orientations for each pair of duplicates. (E) Correlation of the log2-fold changes of protein abundance within paralog pairs estimated from the βm and βp with experimentally measured differences in protein abundance [69, 71, 72]. The estimated protein abundance fold changes were computed using two sets of experimental measurements of protein decay rates [69, 70] and compared to each of four sets of protein abundances. Correlations obtained for the reported βp rates (left) as well as for randomly shuffled βp rates (right) are shown. (TIF) [file pgen.1010756.s001.tif]

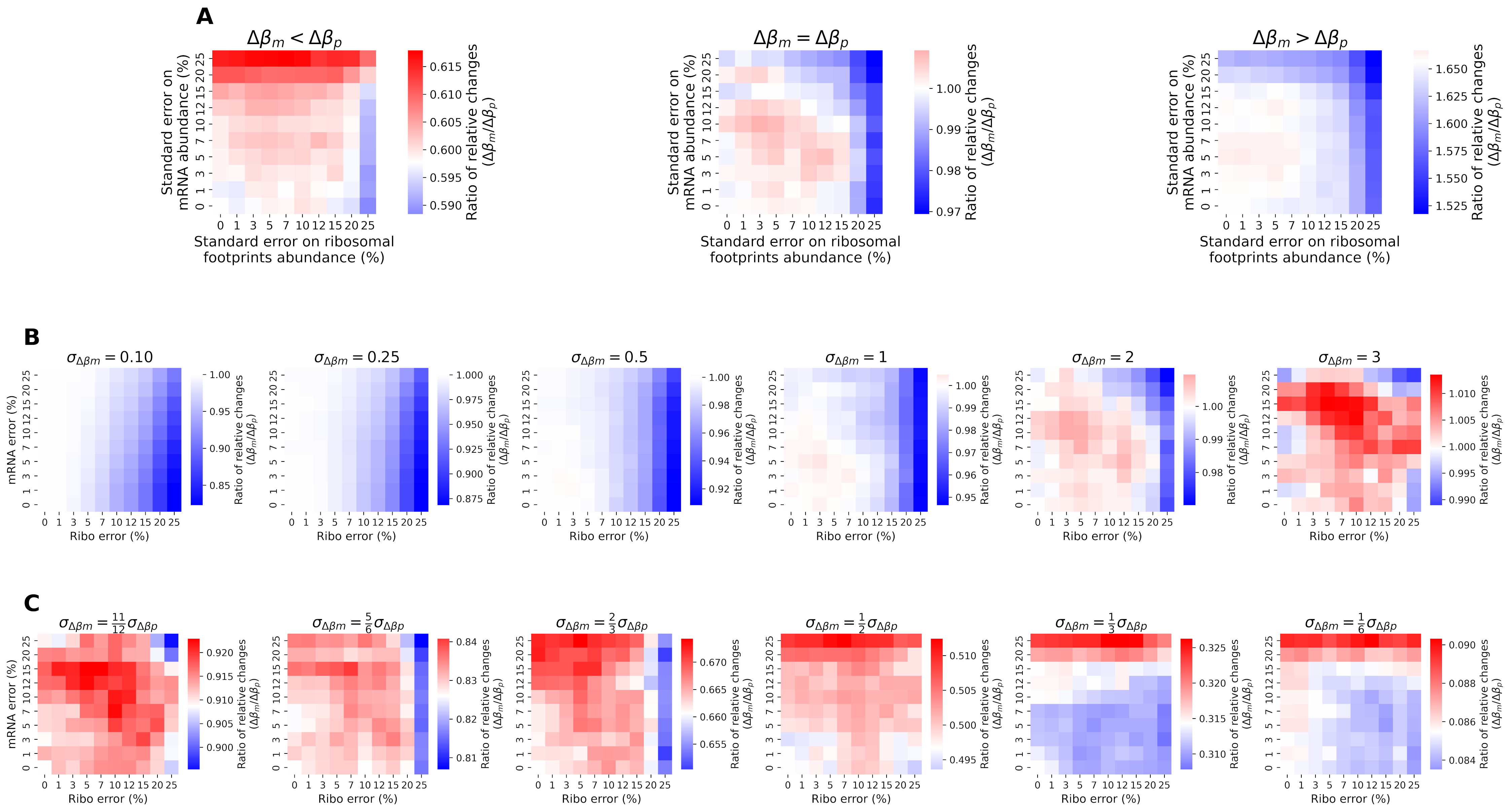

Supplement: S2 Fig — Transcription rates βm and translation rates βp were computed from simulated noisy measurements for randomized gene pairs. The median ratio of transcriptional over translational changes was calculated across all pairs from the noisy measurements, and compared to the ratio obtained from the underlying true βm and βp (center of the color scale on each heatmap). A ratio of 1 indicates equal magnitudes of transcriptional and translational changes. (A) When the contribution of transcription to expression divergence is equal (middle) or greater (right) than that of translation, noise only decreases the ratio and the relative impact of transcription changes is underestimated. An overestimation of the ratio—and thus of the importance of transcription—can occur when translation divergence dominates (left), but its impact is small. The respective standard deviations of the distributions of βm and βp log2-fold changes (σΔβm and σΔβp) have been set according to our estimates of relative divergence. When transcription is assumed to dominate (right, as in the dataset from [25]), these standard deviations are ∼ 2.29, ∼ 1.11, and vice versa if translational divergence is assumed to be larger (left). Identical contributions of transcription and translation changes (center) are modeled as equal standard deviations resulting in the same total variance. (B) When varying the magnitudes of relative divergence but keeping the transcriptional and translational contributions equal (σΔβm = σΔβp), noise still mostly results in an underestimation of the contribution of transcription changes. If the the total variance of the log2-fold changes is much larger than empirically (right; σΔβm = 3), the transcriptional contribution can be overestimated, but the difference is negligible. (C) When translational divergence is assumed to predominate, noise is associated with an overestimation of the contribution of transcription to expression divergence. This overestimation is small—even if the relative m [file pgen.1010756.s002.tif]

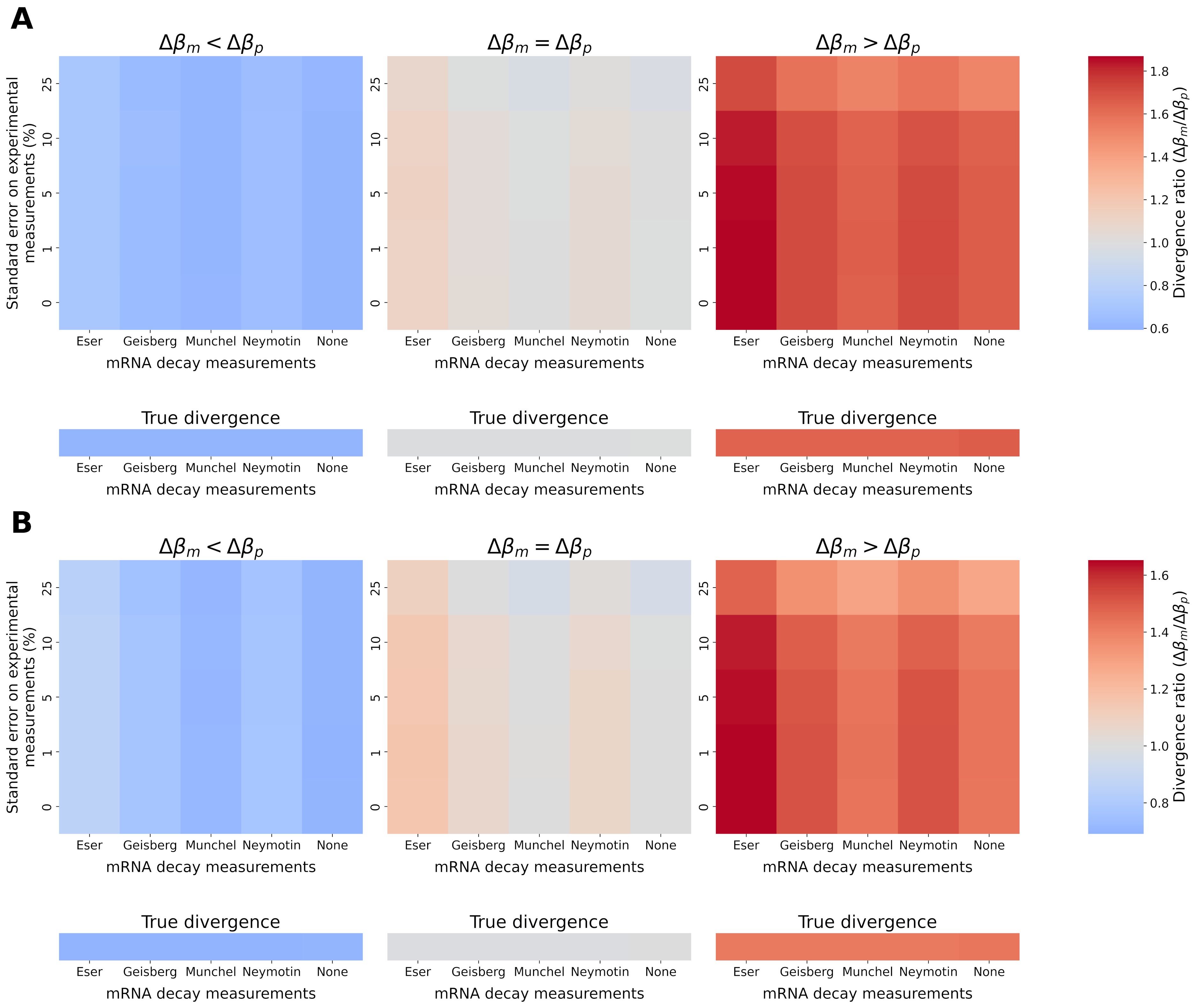

Supplement: S3 Fig — Median ratios of βm/βp relative divergence (value of 1 when both are equal) are shown for randomized gene pairs (n = 10, 000), across a range of noise levels (equal at the transcriptional and translational levels) and experimentally-informed distributions of mRNA decay rate. Transcription and translation log2-fold changes were sampled from distributions with respective standard deviations σΔβm and σΔβp for each simulated pair, while log2-fold changes of decay rate were sampled from the corresponding datasets. The “None” column indicates simulated paralog pairs with invariable mRNA decay. The top heatmaps present the apparent ratios, when failing to account for any gene-to-gene variation in transcript decay, while the bottom single-row heatmaps show the true ratios. (A) Using the standard deviations of transcriptional and translational log2-fold changes obtained from the βm and βp rates reported by [25]. The different scenarios of true divergence (e.g. equal magnitudes of transcriptional and translational divergence, middle) correspond to the same standard deviations σΔβm and σΔβm as in S2A Fig. (B) Assuming halved variances σΔβm2 and σΔβp2 compared to panel A, while still using empirical measurements of mRNA decay. (TIF) [file pgen.1010756.s003.tif]

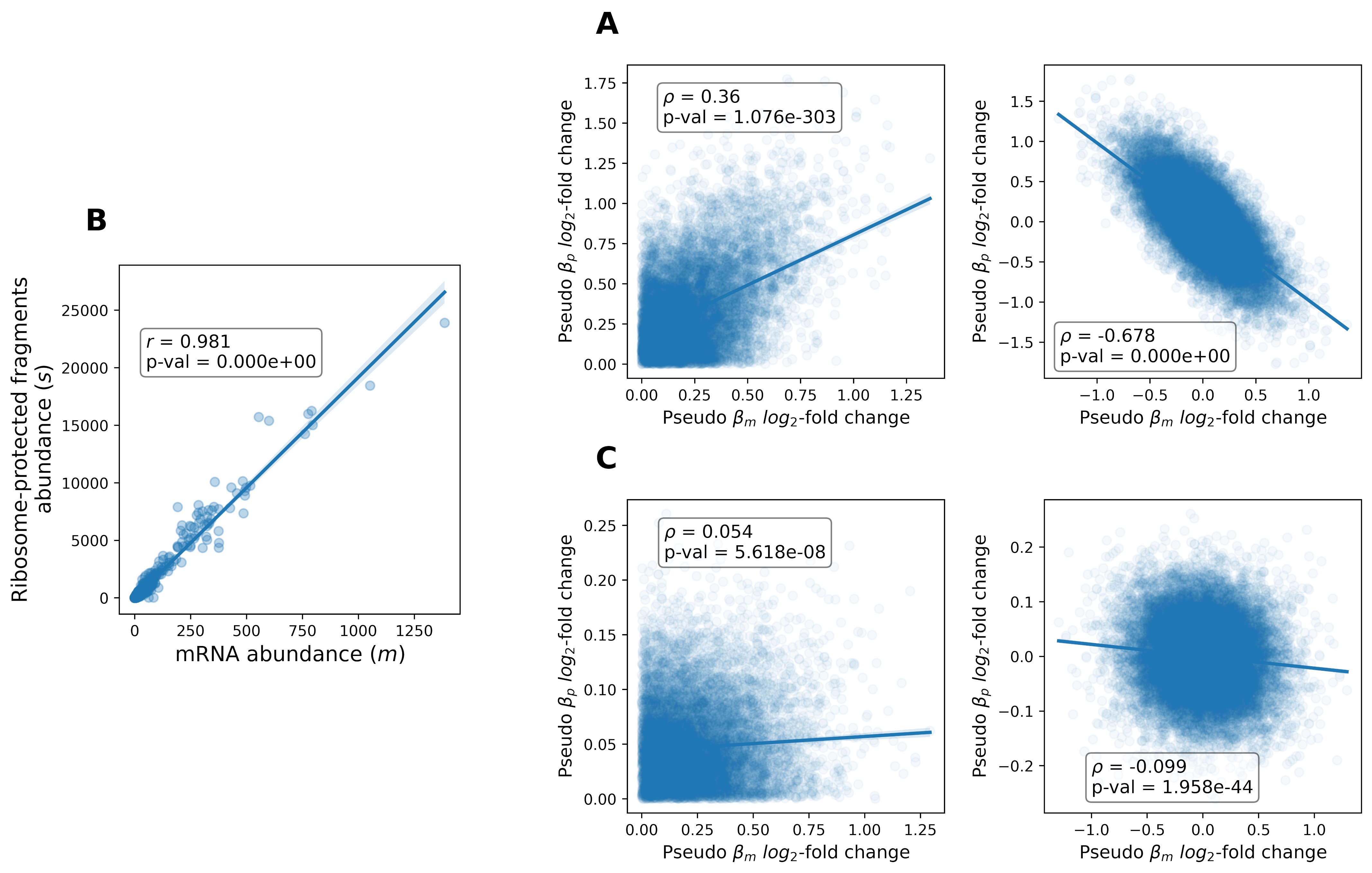

Supplement: S4 Fig — Distributions of pseudo βm and βp log2-fold changes have been computed from random variables (n = 10, 000) mimicking mRNA abundance m and ribosome footprints abundance s. Pseudo βm is set as m, while pseudo βp is sm. (A) Expected correlations for the strictly positive (left) and signed (right) log2-fold changes within random pairs when m and s are independent. (B) Correlation between mRNA abundance m and ribosome footprints abundance s in the data from [30] used in the calculation of the reported βm and βp rates for yeast genes [25]. (C) Expected correlations for the strictly positive (left) and signed (right) log2-fold changes when m and s are strongly correlated as seen in B. (TIF) [file pgen.1010756.s004.tif]

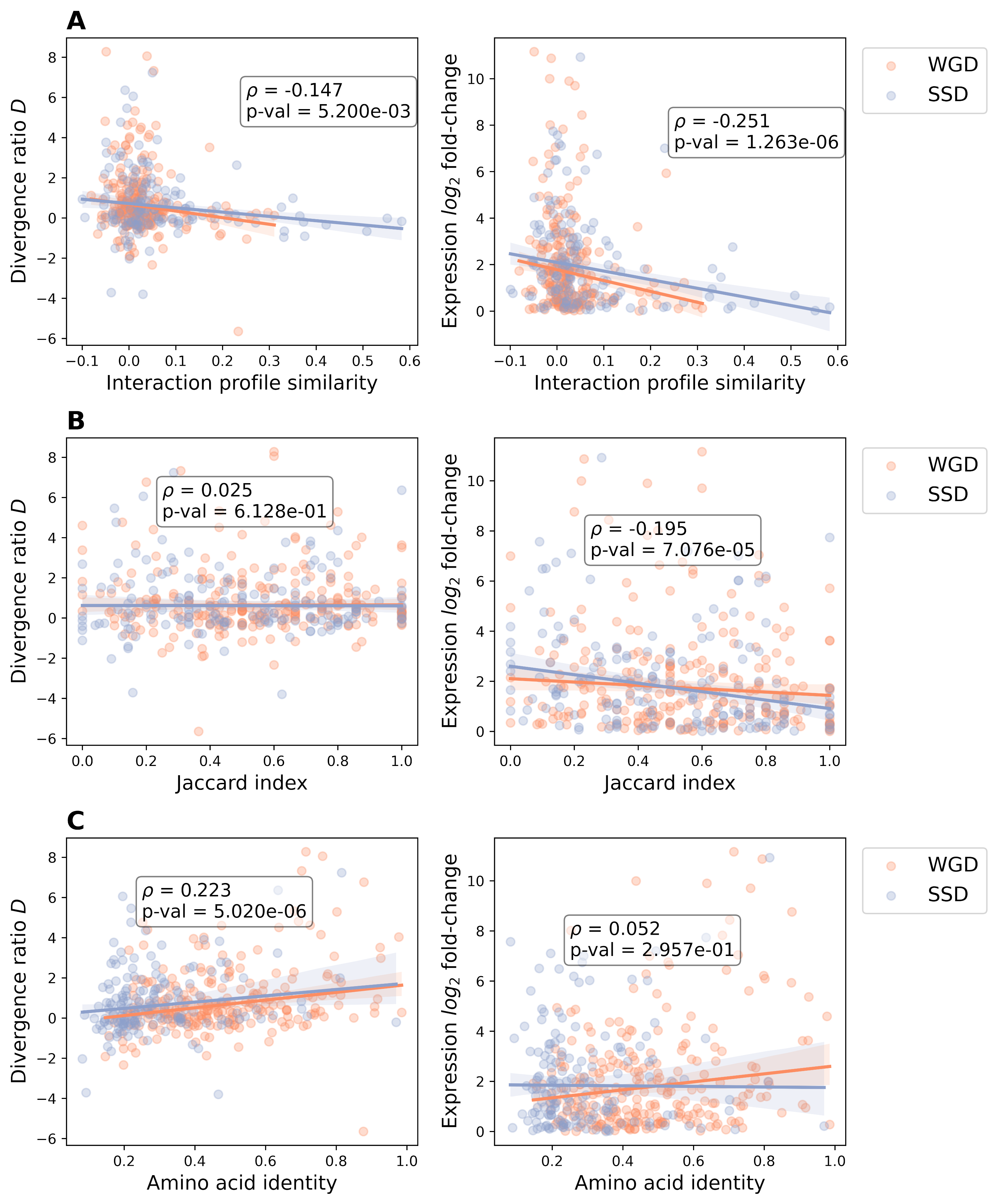

Supplement: S5 Fig — Correlation of the divergence ratio (left), for which positive values indicate a relatively larger divergence in transcription (Methods), or the global expression log2-fold change (combined effect of transcription and translation; right) with three proxies of functional divergence within paralog pairs, separately for WGD- and SSD-derived ones. Spearman’s ρ and p-value were calculated for all duplicate pairs combined. (A) Absolute value of the similarity of genetic interactions profiles within each paralog pair [73]. (B) Overlap (Jaccard index) of GO Slim annotations between paralogs of the same pair. (C) Amino acid identity between the two members of each duplicate pair. (TIF) [file pgen.1010756.s005.tif]

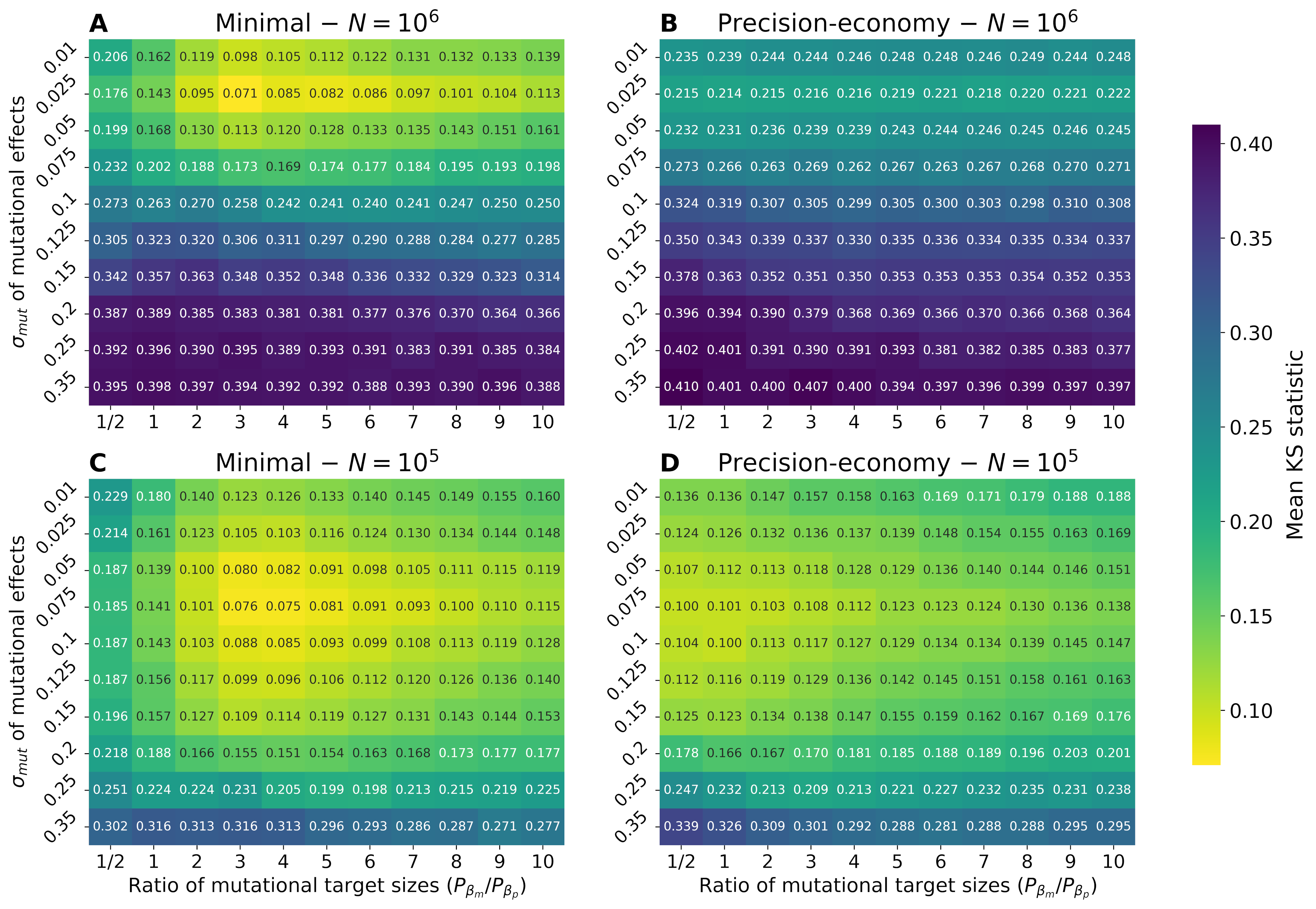

Supplement: S6 Fig — Mean Kolmogorov-Smirnov statistics for the comparisons between empirical and simulated relative divergence distributions are shown. Each value is the grand mean for the three parameters (βm, βp, and P) across three replicate simulations of 2500 randomly generated paralog pairs, when compared to yeast WGD-derived paralogs. (A) Minimal model and high selection efficacy. (B) Precision-economy model and high selection efficacy. (C) Minimal model and reduced efficacy of selection. (D) Precision-economy model and reduced selection efficacy. The smallest grand mean KS statistic across A and B is obtained for σmut = 0.025 (minimal model with Pβm/Pβp = 3), which is accordingly defined as the most realistic value. For C and D, the minimum value is obtained for σmut = 0.075 (minimal model with Pβm/Pβp = 3), which is thus the best-fitting value when a lower selection efficacy is assumed. (TIF) [file pgen.1010756.s006.tif]

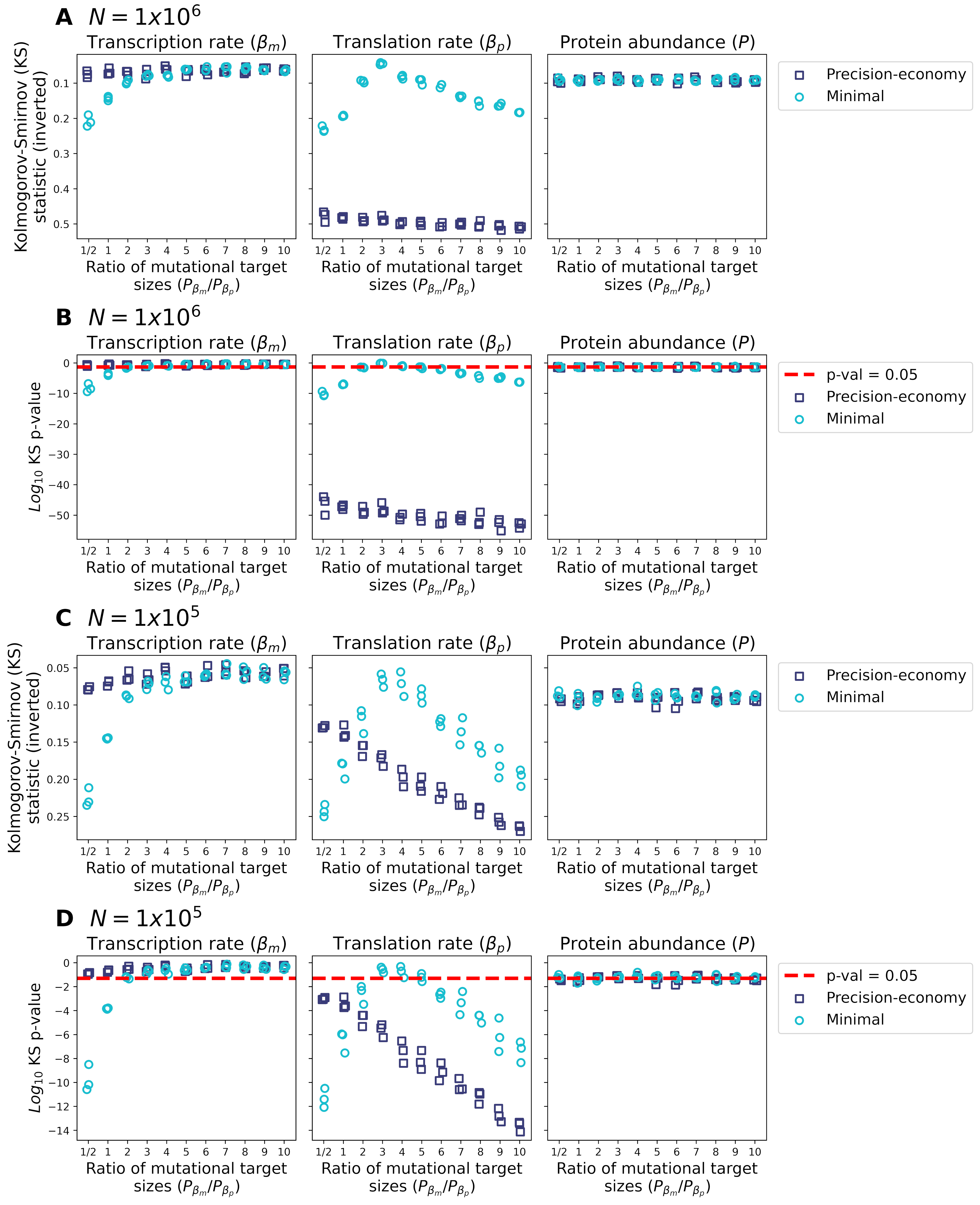

Supplement: S7 Fig — Both models can generate realistic distributions for all three levels, but the precision-economy model is unable to replicate the observed translational divergence under high selection efficacy. Same simulations as shown in Fig 4, with σmut = 0.025 for N = 106 and σmut = 0.075 for N = 105, respectively. (A) Kolmogorov-Smirnov statistics for the three replicate simulations of 2500 randomly generated paralog pairs. (B) Corresponding log10-transformed p-values of the Kolmogorov-Smirnov test. The red dashed line indicates the threshold above which differences are not significant. (C) As in A. (D) As in B. (TIF) [file pgen.1010756.s007.tif]

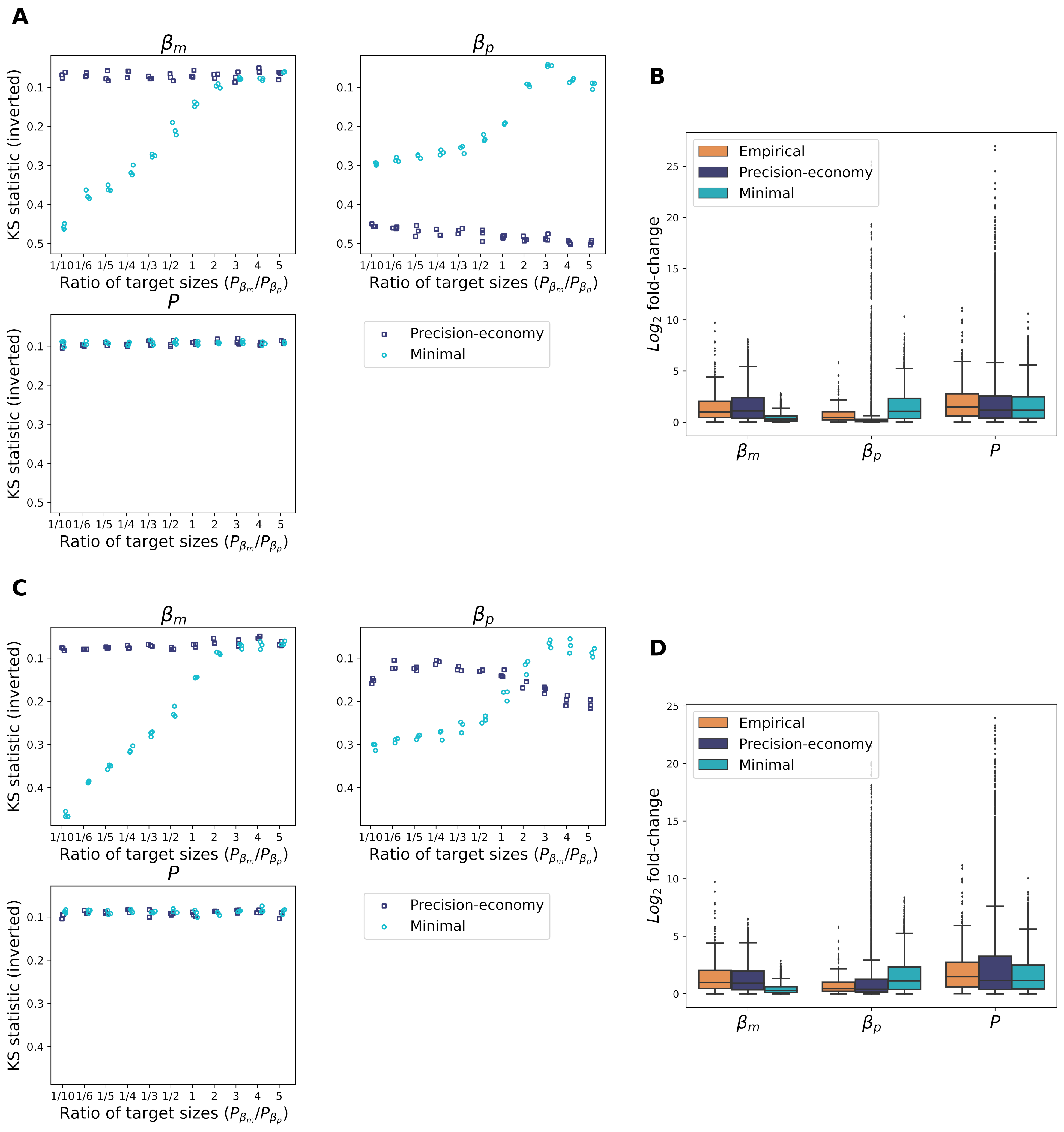

Supplement: S8 Fig — (A) Kolmogorov-Smirnov statistics comparing the fit between empirical (WGD-derived paralogs) and simulated relative divergence distributions for transcription rate (βm), translation rate (βp) and protein abundance (P) for three replicate simulations of 2500 randomly generated paralog pairs when selection efficacy is high (N = 106) and σmut = 0.025 is postulated. (B) Empirical divergence of WGD-derived yeast paralogs compared with the final simulated divergence in transcription, translation and protein abundance for simulations under the minimal and precision-economy models when the mutational target size of translation is ten times larger than that of transcription and selection efficacy is high (same simulations as for Pβm/Pβp = 1/10 on panel A). (C) Same as A, but for simulations with N = 105 and σmut = 0.075. (D) Same comparisons as in B, but performed for the simulations with Pβm/Pβp = 1/10 shown on panel C. When selection efficacy is high as well as when it is reduced, it is the minimal model which reaches the better fit (lower KS statistic) to the distribution of relative translational divergence. (TIF) [file pgen.1010756.s008.tif]

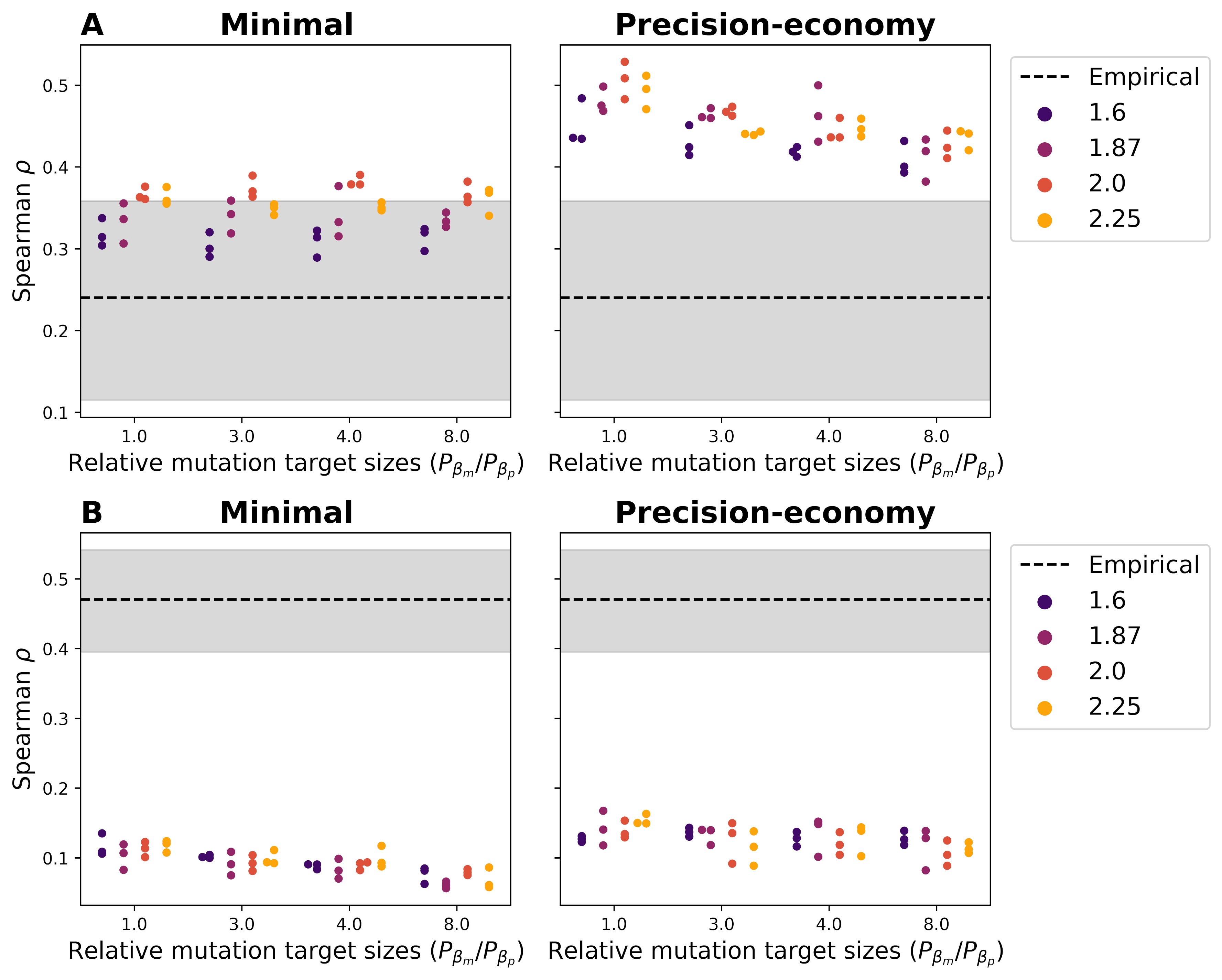

Supplement: S9 Fig — Simulated divergence correlations for three replicate simulations of 2500 randomly generated paralog pairs under the minimal and precision-economy models for a sample of mutational target size ratios and a range of post-duplication variation Δopt of the cumulative protein abundance optimum, when the efficacy of selection is assumed to be high (N = 106; σmut = 0.025). A Δopt of 2.0 indicates that the doubling of protein abundance resulting from the duplication event is perfectly optimal. All simulations stopped and evaluated according to the empirical expression divergence of yeast WGD-derived duplicate pairs, as in Fig 4. The dashed lines and the shaded areas represent the empirical value of the corresponding correlation and its 95% confidence interval. (A) Correlation between the magnitudes of the log2-fold changes in transcription and translation. (B) Correlation between the signed log2-fold changes in transcription and translation. (TIF) [file pgen.1010756.s009.tif]

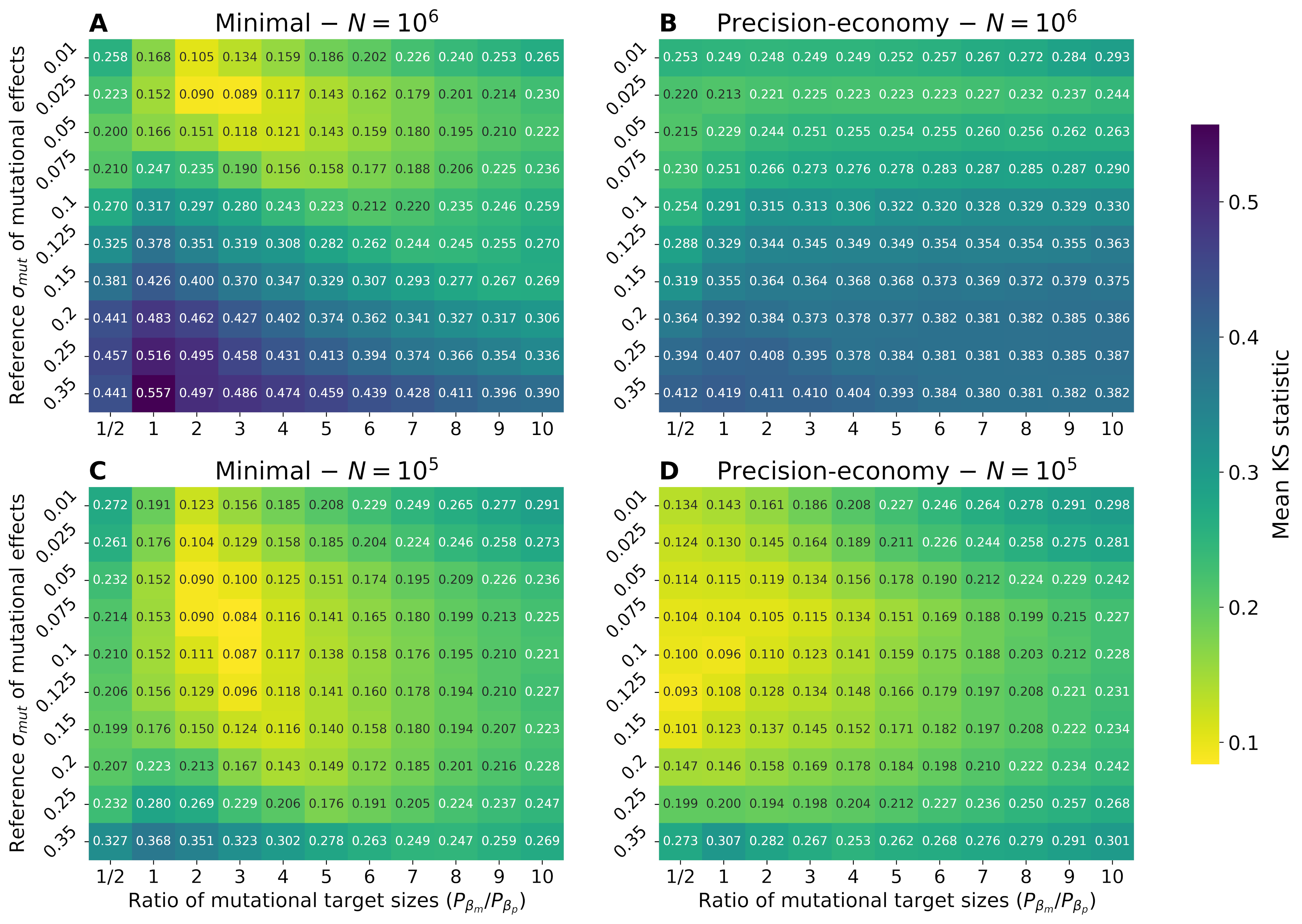

Supplement: S10 Fig — Under this framework, standard deviations σβm and σβp of transcriptional and translational effects are set by the relative mutational target sizes Pβm and Pβp, but their precise values are chosen to result in the same mean change of protein abundance per mutation as a reference σmut (shown on the figure) in the univariate implementation (Methods). The mean Kolmogorov-Smirnov statistics for the comparisons between empirical and simulated relative divergence distributions are shown. Each value is the grand mean for the three parameters (βm, βp, and P) across three replicate simulations of 2500 randomly generated paralog pairs, performed and assessed according to the WGD-derived paralogs of yeast. (A) Minimal model and high selection efficacy. (B) Precision-economy model and high selection efficacy. (C) Minimal model and reduced efficacy of selection. (D) Precision-economy model and reduced selection efficacy. The overall lowest grand mean KS statistic across A and B is obtained for a reference σmut of 0.025 (minimal model with Pβm/Pβp = 3), indicating that it is the most realistic value. Across C and D, the minimum is obtained for a reference σmut of 0.075 (minimal model with Pβm/Pβp = 3), which is thus similarly the best-fitting value when a lower selection efficacy is assumed. (TIF) [file pgen.1010756.s010.tif]

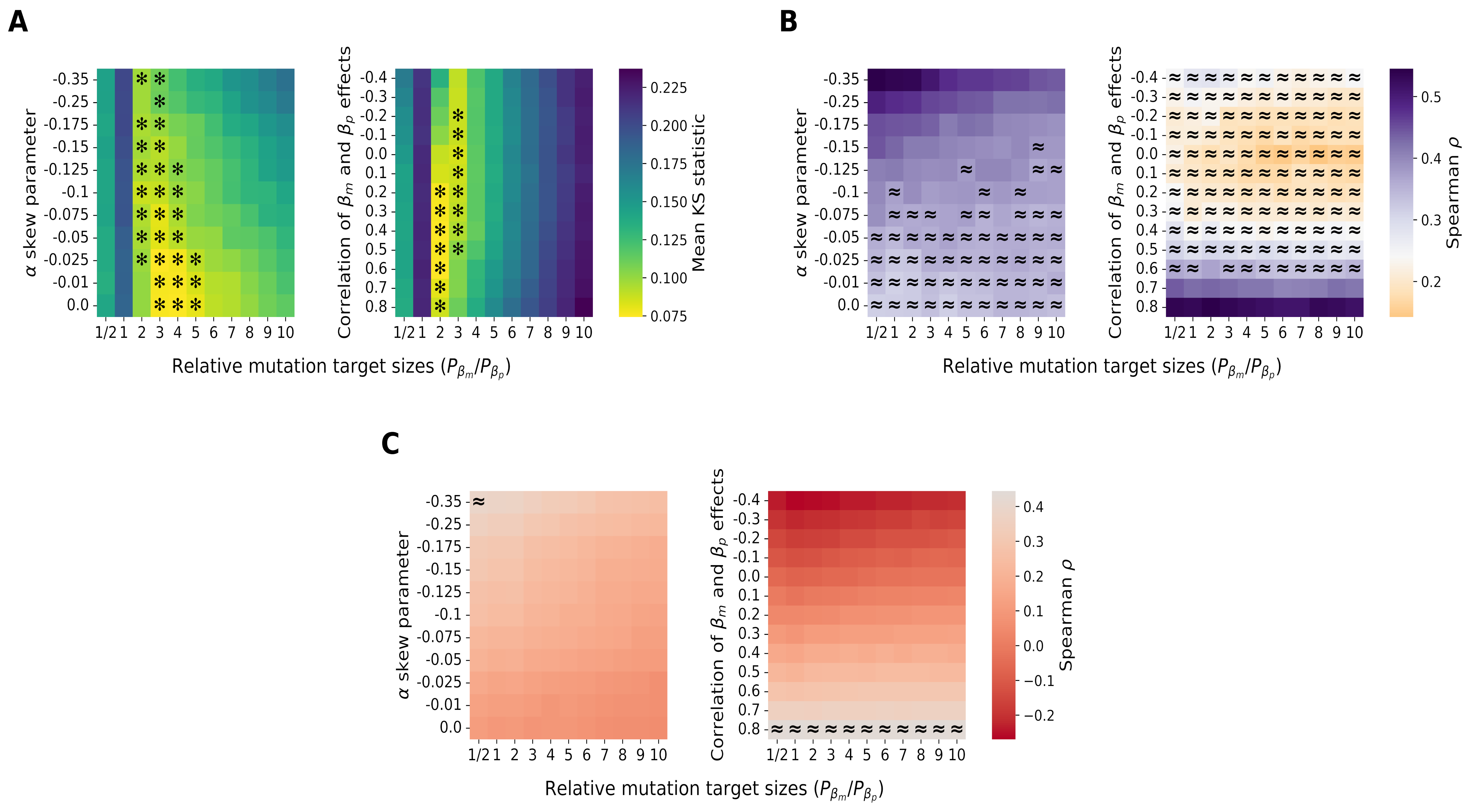

Supplement: S11 Fig — (A) Replication of the three distributions of relative divergence (transcription, translation and protein abundance) for a range of mutational effects distribution asymmetry (left) or correlations between the effects of transcriptional and translational mutations (right), as shown by grand mean KS statistics. Asterisks identify instances where all three magnitudes of relative divergence are realistic for at least one of three replicate simulations (p > 0.05, Mood’s median test). (B, C) Average final correlation across the three replicate simulations between 1) the magnitudes of transcriptional and translational log2-fold changes (in B) and 2) the signed log2-fold changes in transcription and translation (in C) across the same ranges of mutational effects distribution asymmetry or correlations between the effects of transcriptional and translational mutations. In each case, ≈ designates parameter combinations where a correlation coefficient within the 95% confidence interval of the empirical value was obtained for at least one replicate simulation. All corresponding simulations performed as in Fig 5, but for N = 105 and σmut = 0.075. (TIF) [file pgen.1010756.s011.tif]

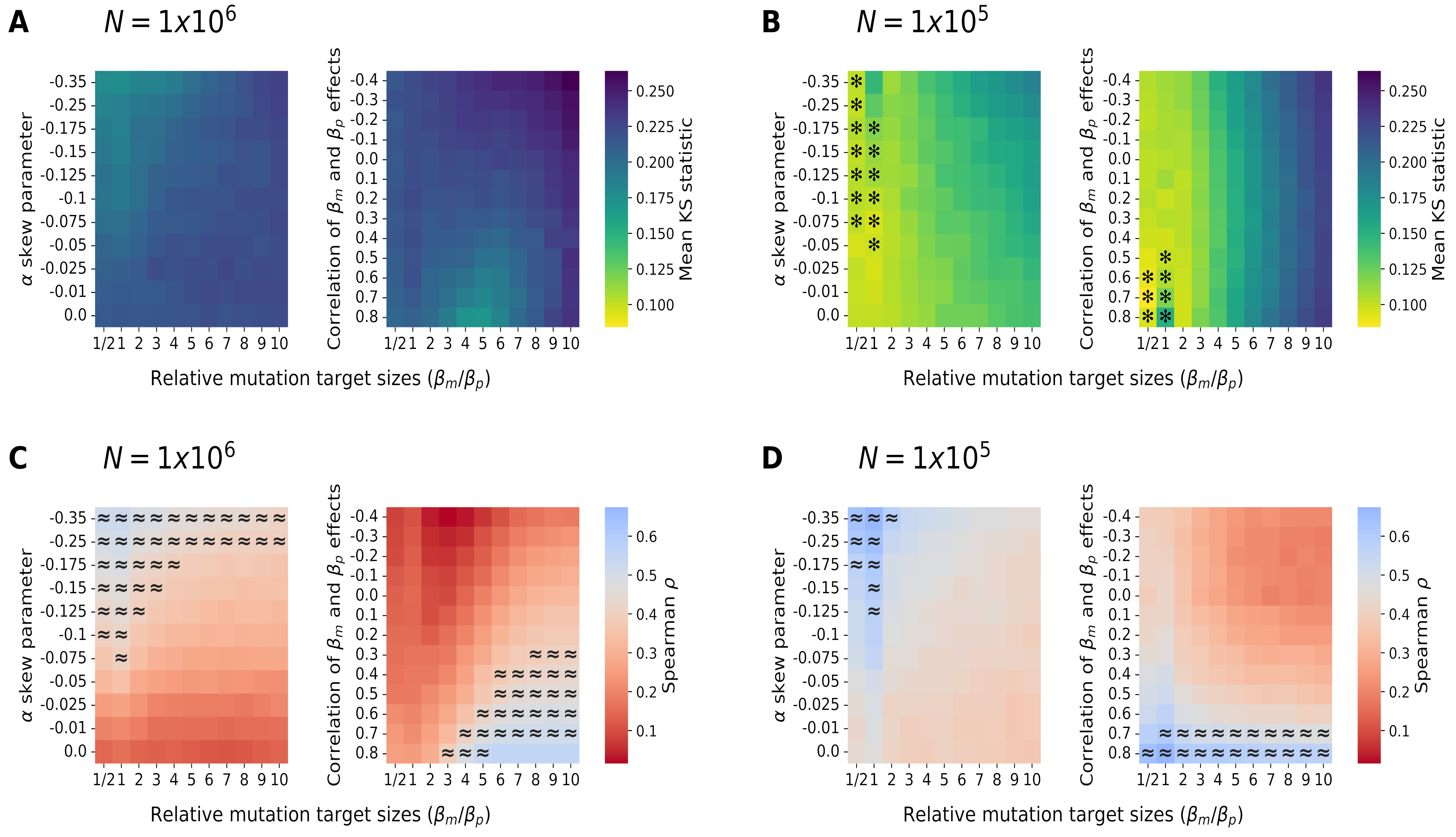

Supplement: S12 Fig — Results are shown for three replicate simulations of 2500 paralog pairs, as in Fig 5 and S11 Fig. (A, B) Average correlation between the magnitudes (log2-fold changes) of relative divergence in transcription and in translation within duplicate pairs, for a range of skewness of mutational effects distribution (left) or of correlations between the βm and βp effects (right). (C, D) Average correlation between the signed magnitudes (signed log2-fold changes) of transcriptional and translational relative divergences along the same ranges of skewness (left) or transcription-translation mutational correlations (right). Across all heatmaps, the symbol ≈ identifies parameter combinations where a correlation coefficient within the 95% confidence interval of the empirical value was obtained for at least one replicate simulation. (TIF) [file pgen.1010756.s012.tif]

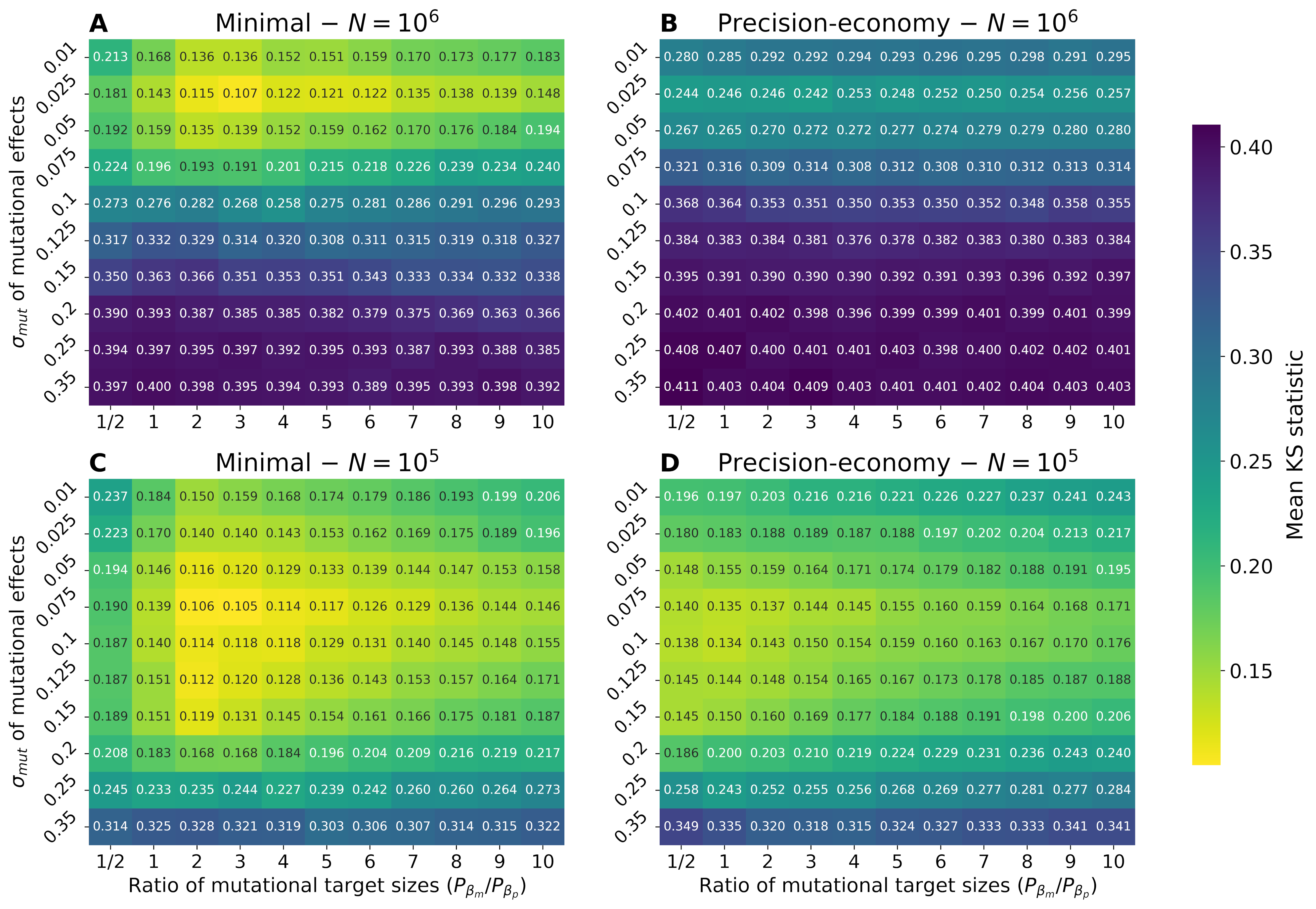

Supplement: S13 Fig — The grand means of Kolmogorov-Smirnov statistics (across βm, βp and P) for three replicate simulations of 2500 randomly generated paralog pairs are shown. Testing of the end conditions of the simulations and comparisons of the resulting divergence patterns were both done according to the SSD-derived duplicate pairs of S. cerevisiae. (A) Minimal model and high selection efficacy. (B) Precision-economy model and high selection efficacy. (C) Minimal model and reduced efficacy of selection. (D) Precision-economy model and reduced selection efficacy. (TIF) [file pgen.1010756.s013.tif]

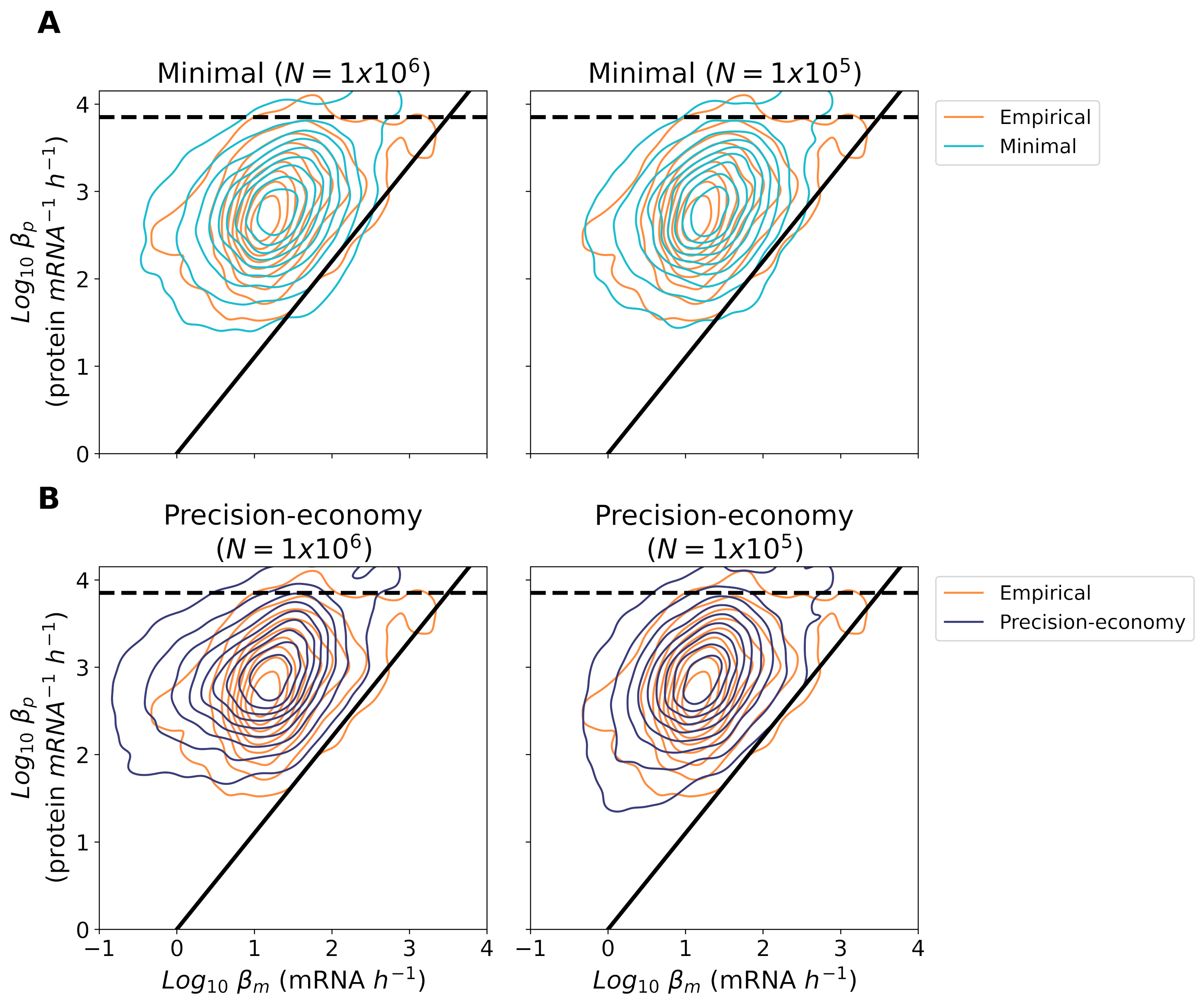

Supplement: S14 Fig — Comparisons of the empirical distribution of yeast genes in transcription (βm) and translation (βp) rates [25] with the distributions of simulated paralogs obtained from the combination of three replicate experiments of in silico evolution with 2500 randomly generated duplicate pairs (selected simulations from Fig 4). The simulations resulting in the best-fitting patterns of expression divergence (as previously assessed in S6 Fig) are shown in each case. The dashed line represents the estimated maximal translation rate [25], while the diagonal is the boundary of the depleted region defined by [25]—below which only 1% of all yeast genes are found. (A) Comparisons with simulations made under the minimal model, under assumptions of high (left; σmut = 0.025 and Pβm/Pβp = 3) and reduced selection efficacy (right; σmut = 0.075 and Pβm/Pβp = 4). (B) Comparisons with simulations made under the precision-economy model for both levels of selection efficacy (left: σmut = 0.025 and Pβm/Pβp = 1; right: σmut = 0.075 and Pβm/Pβp = 1/2). (TIF) [file pgen.1010756.s014.tif]

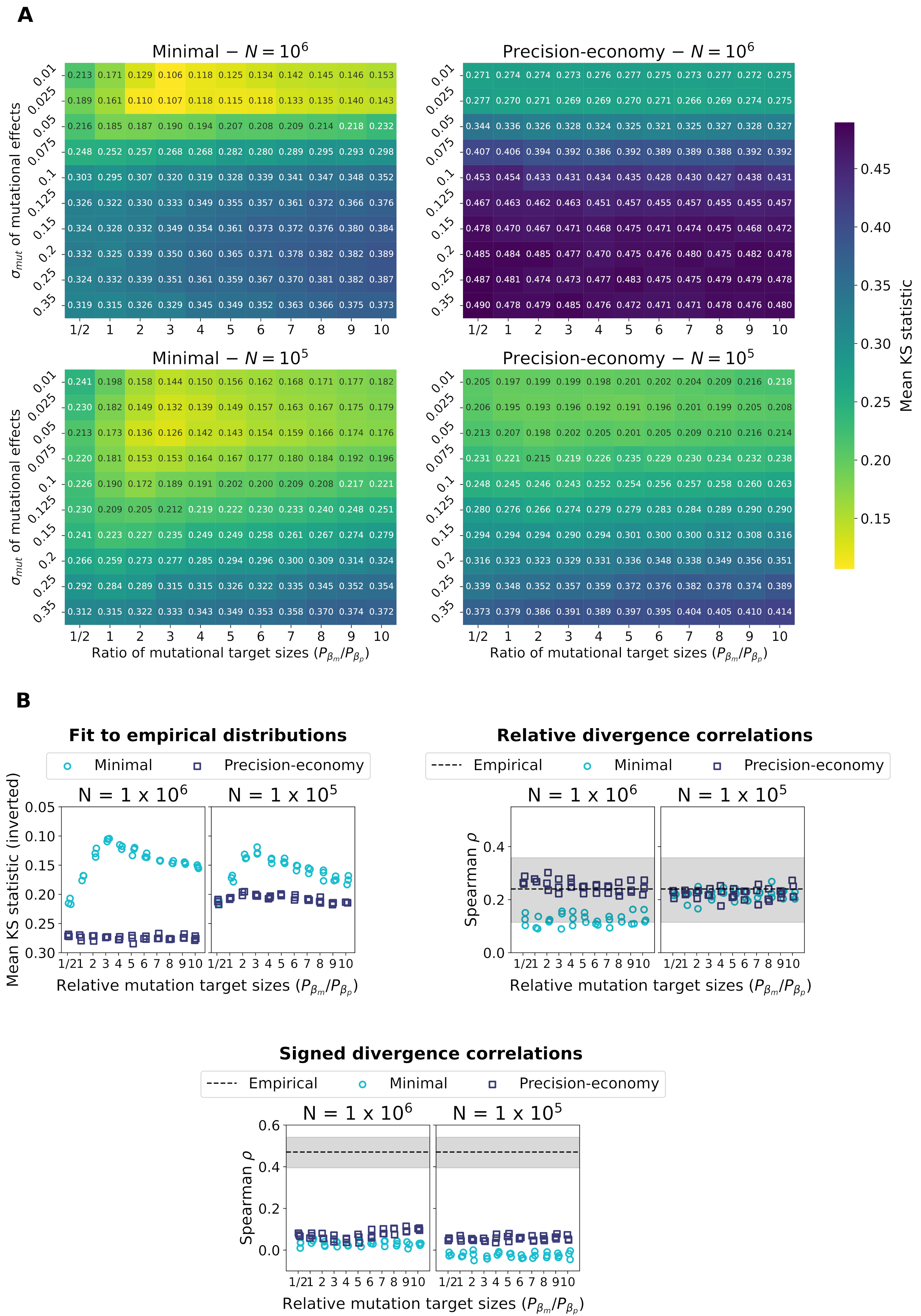

Supplement: S15 Fig — All gene loss events which would have been tolerated by selection at the end of each simulation were performed prior to the calculation of the summary statistics shown. Results for three replicate simulations of 2500 paralog pairs, stopped and evaluated according to yeast WGD-derived duplicates, are shown as previously. (A) Grand means of the Kolmogorov-Smirnov statistics for βm, βp, and P across ranges of standard deviations σmut and mutational target size ratios, as in S6 Fig). (B) Mean KS statistics and divergence correlations across the range of mutational target size ratios for three replicate simulations performed at the best-fitting standard deviations of mutational effects (σmut = 0.01 for N = 106, and σmut = 0.05 for N = 105), as in Fig 4B–4D. Across the latter panel, patterns very similar to those observed in the corresponding figure of the main text are obtained, but the fit to the empirical distributions is slightly reduced (increased mean KS statistics). (TIF) [file pgen.1010756.s015.tif]

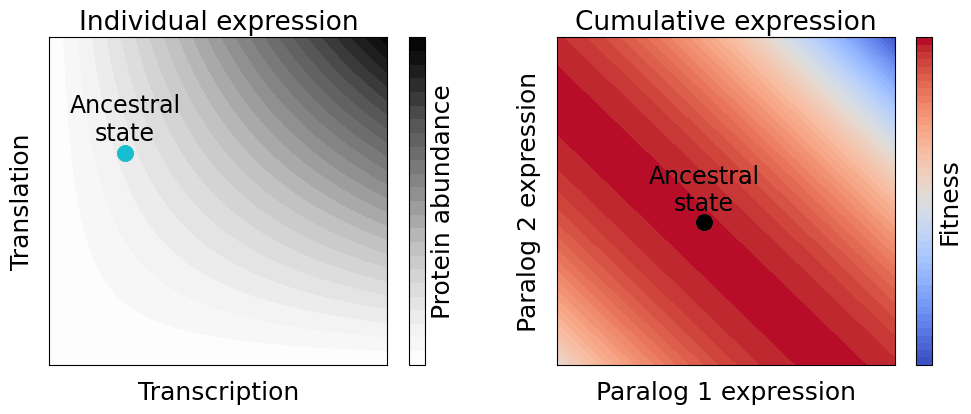

Supplement: S1 File — Mutations cause small changes in the transcription and translation rates of paralogs P1 and P2. Selection to maintain cumulative expression however maintains their total protein abundance at an approximately constant level. (GIF) [file pgen.1010756.s016.gif]
