# Supplementary material for: Evolutionary trade-off and mutational bias could favor transcriptional over translational divergence within paralog pairs
Source: PLoS Genet. 2023 May 26;19(5):e1010756. doi: 10.1371/journal.pgen.1010756 (PMC10275480; doi:10.1371/journal.pgen.1010756)
Supplement: S1 Methods — (PDF) [file pgen.1010756.s017.pdf]

Supplementary Information
Evolutionary trade-off and mutational bias could favor transcriptional over translational divergence within paralog pairs

Simon Aubé[1,2,3,4*], Lou Nielly-Thibault[2,3,4,5,6], Christian R. Landry[1,2,3,4,5*]

1 Département de biochimie, de microbiologie et de bio-informatique, Faculté des sciences et de génie, Université Laval, Québec, Québec, Canada
2 Institut de Biologie Intégrative et des Systèmes, Université Laval, Québec, Québev, Canada
3 PROTEO, Le regroupement québécois de recherche sur la fonction, l'ingénierie et les applications des protéines, Université Laval, Québec, Québec, Canada
4 Centre de Recherche en Données Massives, Université Laval, Québec, Québec, Canada
5 Département de biologie, Faculté des sciences et de génie, Université Laval, Québec, Québec, Canada
6 Current address: Département de médecine moléculaire, Faculté de médecine, Université Laval, Québec, Québec, Canada

* simon.aube.2@ulaval.ca; Christian.Landry@bio.ulaval.ca

Extended Methods

Defining gene-specific fitness functions

We defined the relationship between fitness and the expression level (protein abundance) of any gene using parabolic functions. Each such function $W(p)$ is described by its vertex (p_{opt}, μ), where the highest fitness is obtained for an optimal protein abundance, and a noise sensitivity Q [1] related to the curvature $W''(p_{opt})$ of the parabola (Eq 1).

$$Q = -\frac{1}{2} W''(p_{opt})\, p_{opt} \tag{1}$$

Since the curvature of the function can be isolated from the previous equation, Q can be used to compute the a parameter of the parabola, and from there, obtain the full equation of the standard form $ax^2 + bx + c$:

$$\begin{aligned} a &= \frac{W''(p_{opt})}{2} = \frac{-2\left(\frac{Q}{p_{opt}}\right)}{2} = -\frac{Q}{p_{opt}} \\ b &= -2ap_{opt} = -2\left(-\frac{Q}{p_{opt}}\right)p_{opt} = 2Q \\ c &= \mu - ap_{opt}^2 - bp_{opt} = \mu - Qp_{opt} \end{aligned} \tag{2}$$

Following a duplication event, the new fitness function of cumulative protein abundance $W(p_1 + p_2)$ is defined from the $W(p)$ function of the ancestral gene. Only one parameter is modified, p_{opt}, which is multiplied by 1.87 (see below). The post-duplication fitness function thus becomes:

$$W(p_1 + p_2) = -\frac{Q}{1.87p_{opt}}(p_1 + p_2)^2 + 2Q(p_1 + p_2) + (\mu - Q(1.87p_{opt})) \tag{3}$$

Selecting the post-duplication change in optimal protein abundance

After a gene duplication event, the total transcription of the resulting gene pair is the ancestral transcription rate of the singleton times factor Δ_m. Similarly, the optimal cumulative protein abundance for the two paralogs is Δ_{opt} times the original optimal expression p_{opt} of the ancestral singleton. From equation Eq 3, a strictly positive post-duplication fitness is thus obtained when:

$$-\frac{Q}{\Delta_{opt}p_{opt}}(\Delta_m p_{opt})^2 + 2Q(\Delta_m p_{opt}) + \mu - Q\Delta_{opt}p_{opt} > 0 \tag{4}$$

Multiplying by Δ_{opt}, an expression of the form $a\Delta_{opt}^2 + b\Delta_{opt} + c$ is obtained:

$$\begin{aligned} &-\frac{Q}{p_{opt}}(\Delta_m p_{opt})^2 + 2Q(\Delta_m p_{opt})\Delta_{opt} + \mu\Delta_{opt} - Q\Delta_{opt}^2 p_{opt} > 0 \\ &\Rightarrow -Qp_{opt}\Delta_{opt}^2 + (2Q\Delta_m p_{opt} + \mu)\Delta_{opt} - Q\Delta_m^2 p_{opt} > 0 \end{aligned} \tag{5}$$

To solve for Δ_{opt}, we consider the most extreme case: an ancestral gene with the highest possible noise sensitivity and protein abundance. Accordingly, Q is set to the highest possible value within the framework of [1] ($\sim 6.8588 \times 10^{-6}$) and p_{opt} is set to the highest expression level observed in the dataset ($\sim 6.0649 \times 10^{6}$ proteins per cell).

Constant Δ_m is set to 2, meaning a doubling of total transcription, and a maximum growth rate μ of $0.42h^{-1}$ is considered. The following bounds are obtained:

$$\sim 1.87 < \Delta_{opt} < \sim 2.15 \tag{6}$$

In accordance with this result, we used $\Delta_{opt} = 1.87$ throughout the current work. For all the random seeds used in the simulations, this value, obtained for the minimal model, was also valid for the precision-economy model.

Estimating expression noise for a protein expressed from a pair of paralogous genes

As mentioned in the main text, the variance of protein abundance for a single-copy gene can be estimated as:

$$\sigma^2 \approx p^2 \left(\frac{1}{p} + \frac{\alpha_p}{\beta_m} + c_{v0}^2 \right)$$

In order to obtain a similar equation for a pair of identical paralogs expressing the same protein, the extrinsic and intrinsic components of noise must be treated separately. Because two duplicate genes are by definition present in the same cell, extrinsic fluctuations will be equal for both of them (as we assume they are identical and thereby share all regulators), while intrinsic fluctuations will independently affect the expression level of each copy. As it does not depend on any gene-specific property, the noise floor c_{v0} is chosen as the extrinsic component (Eq 7). Although recent modeling work indicates that this noise floor is extrinsic in nature [2], we note that it might still not fully represent extrinsic noise.

$$\sigma^2 = \sigma_{int}^2 + \sigma_{ext}^2 \approx p^2 \left(\frac{1}{p} + \frac{\alpha_p}{\beta_m} \right) + p^2 c_{v0}^2 \tag{7}$$

The variance on the cumulative protein abundance of P_1 and P_2 can be obtained from the variances of their individual protein abundances. In order to perform this calculation, the fluctuations from mean protein abundance across a population of cells can be seen as a random variable with mean 0 and variance σ^2. As shown above (Eq 7), this random variable is itself the sum of two other random variables representing the intrinsic and extrinsic components of these fluctuations. In turn, these two components are each a sum of the respective contributions of both paralogs. For intrinsic noise, the cumulative variance is the sum of the intrinsic variances respectively calculated for each duplicate gene. By definition, intrinsic fluctuations are uncorrelated between duplicates, meaning that the intrinsic components are two independent variables and that their variances can be summed. In contrast, extrinsic fluctuations are the same for two identical paralogs within the same cell, resulting in the extrinsic components of protein abundance variance for P_1 and P_2 being two perfectly positively correlated variables. Their cumulative variance is thus the square of the sum of their standard deviations. Accordingly, the variance of cumulative protein abundance for a duplicate couple is obtained using the following equation:

$$\begin{aligned} \sigma_{tot}^2 &= \sigma_{int1}^2 + \sigma_{int2}^2 + (\sigma_{ext1} + \sigma_{ext2})^2 \\ &\approx p_1^2 \left(\frac{1}{p_1} + \frac{\alpha_p}{\beta_{m1}} \right) + p_2^2 \left(\frac{1}{p_2} + \frac{\alpha_p}{\beta_{m2}} \right) + (p_1 c_{v0} + p_2 c_{v0})^2 \end{aligned} \tag{8}$$

Selection of valid ancestral genes

During the generation of ancestral singletons, a minimal threshold of fitness function curvature is enforced. This ensures that all selected genes are sensitive enough to

changes in protein abundance for the immediate post-duplication loss of a paralog to be deleterious. A duplicate pair for which this would not be the case would rapidly revert to the singleton state.

Classical population genetics theory indicates that a mutation needs to cause a loss of fitness greater than the inverse of the effective population size to be efficiently selected against. Accordingly, we want to identify conditions under which the loss of a paralog immediately after duplication would reduce fitness by more than $1/N$. That is:

$$W\left(p_{tot}\right) - W\left(\frac{p_{tot}}{2}\right) > \frac{1}{N} \tag{9}$$

For the filtering of singleton genes, it is more convenient to express p_{tot} as twice the ancestral protein abundance optimum p_{opt}. Using the parabola of form $ap_{tot}^2 + bp_{tot} + c$ that is the fitness function $W\left(p_{opt}\right)$ and adding constants Δ_m and Δ_{opt} – describing the post-duplication change of total transcription and optimal cumulative protein abundance, respectively – to generalize to any duplication, we obtain:

$$\begin{aligned} &\left(-\frac{Q}{\Delta_{opt}p_{opt}}\left(\Delta_m p_{opt}\right)^2 + 2Q\left(\Delta_m p_{opt}\right)\right) - \\ &\left(-\frac{Q}{\Delta_{opt}p_{opt}}\left(\frac{\Delta_m p_{opt}}{2}\right)^2 + 2Q\left(\frac{\Delta_m p_{opt}}{2}\right)\right) > \frac{1}{N} \end{aligned} \tag{10}$$

Summing and simplifying, we obtain the following expression:

$$\Delta_m Q p_{opt}\left(1 - \frac{3\Delta_m}{4\Delta_{opt}}\right) > \frac{1}{N} \tag{11}$$

Accordingly, all ancestral singletons included in the current simulations combine a noise sensitivity Q and a protein abundance optimum p_{opt} which satisfy the following condition:

$$Qp_{opt} > \frac{1}{c_n N} \tag{12}$$

$$where\ c_n = \Delta_m\left(1 - \frac{3\Delta_m}{4\Delta_{opt}}\right)$$

This condition is only valid when $c_n > 0$, which implies that $\frac{3\Delta_m}{4\Delta_{opt}} < 1$. In accordance with this, all simulations presented in the current work are done under $\frac{3}{4}\Delta_m < \Delta_{opt}$.

References

1. Hausser J, Mayo A, Keren L, Alon U. Central dogma rates and the trade-off between precision and economy in gene expression. Nature Communications. 2019;10(1):68. doi:10.1038/s41467-018-07391-8.

2. Jedrak J, Ochab-Marcinek A. Contributions to the 'noise floor' in gene expression in a population of dividing cells. Scientific Reports. 2020;10(1):13533. doi:10.1038/s41598-020-69217-2.
