# Supplementary material for: Evolutionary trade-off and mutational bias could favor transcriptional over translational divergence within paralog pairs
Source: PLoS Genet. 2023 May 26;19(5):e1010756. doi: 10.1371/journal.pgen.1010756 (PMC10275480; doi:10.1371/journal.pgen.1010756)
Supplement: Attachment — Submitted filename: Response_reviews_SA.pdf [file pgen.1010756.s018.pdf]

Response to reviewer comments

We thank all three reviewers for their comments, which we think helped us greatly improve our manuscript.

Reviewer 1:

1. *The main observation that transcription rates are more divergent than translation rates between paralogs makes perfect sense based on our current knowledge of these processes. However, because the actual difference is relatively small (Fig 1A) and because this is a central observation of this paper, I would encourage the authors to investigate potential confounding factors very carefully. One possible such confounding factor is the precision of measurements for transcription and translation rates. If the measures of transcription rates tend to be noisier than those of translation rates, it could artificially inflate the observed contribution of transcription rates to the divergence of expression between duplicates. While I do not know enough about these methods to tell if one is more precise than the other, I did notice in Hausser 2019 (the main dataset used in this paper) that translation rates tend to be higher than transcription rates (see Figure 2a of Hausser 2019, where a typical gene producing 10 transcripts per hours would have a translation rates (proteins per mRNA per hour) between 100 and 1,000 – the yellow area in the graph). Assuming that measuring a rare phenomenon is noisier than measuring a frequent phenomenon, it seems plausible that transcription rates measurements are noisier than translation rates. If biological replicates are available in the original study measuring transcription and translation rates in yeast, the authors could use these replicates to estimate the level of noise in each of these two measurements and estimate its contribution to the pattern reported in this manuscript.*

We thank the reviewer for this suggestion. As this observation is central to our paper, it is indeed relevant to investigate more thoroughly how it may be confounded by experimental variation.

While this was not sufficiently clear from our article, the underlying measurements for transcription are not related to a much rarer phenomenon than those for translation. This is because both rates are calculated from the (normalized) number of reads obtained for each gene from a paired experiment of mRNA-seq and ribosome profiling. One could even expect the latter measurements – from which the translation rates have been inferred – to be noisier, since ribosome profiling involves sequencing the ribosome-protected fragments of the mRNAs, a subsample of total mRNAs that thus requires further experimental steps. Enriching for this ribosomal fraction likely introduces further experimental variation and also means that only a subset of the initial mRNA is used for the translational measurements.

It is also important to note that, due to how transcription and translation rates have been calculated by Hausser et al. (2019), any noise in the mRNA-seq measurements would affect the estimation of both rates. This is because the estimated mRNA abundance, which is directly calculated from the mRNA-seq measurements, is used in both calculations.

In case this reasoning was overly simplistic, we decided to follow the reviewer's recommendation and assess more rigorously how experimental noise may affect our estimations of relative divergence in transcription and in translation. As the original data from Weinberg et al. (2016)

does not include any biological replicates, we could not measure experimental variation directly. Instead, we used simulations under varying levels of noise to determine how measurement errors could have skewed our results. We randomly sampled β_m and β_p values and inferred the corresponding mRNA and ribosome-protected fragments abundances (the two quantities directly obtained from mRNA-seq and ribosomal profiling normalized read counts), to which we added various levels of gaussian noise (**Methods**). In each case, we compared the exact β_m and β_p log_2-fold changes to the ones obtained from the noisy measurements, which revealed that noise most often led to an underestimation of the contribution of transcriptional changes to expression divergence (**Figs S2-S3**). In the rare instances where the contribution of transcription divergence can be overestimated, it is only by a small magnitude. As such, these additional simulations clearly show that our observation is very unlikely to be an artifact due to experimental noise. We now briefly discuss this in the first subsection of our *Results* (*Yeast duplicates mostly diverged in transcription*), paragraph 5.

2. *In Figure 1D, the authors correlate the divergence D (equation 2) with protein abundance log2 fold-change. While I'm not sure exactly how the protein abundance was measured, it should be equal (for gene 1) to β_{m1} × [mRNA decay constant] × β_{p1} × [protein decay constant]. In other words, both the x and y axes values are directly dependent on β_m and β_p, which could cause a spurious correlation. The author correctly identify this problem and perform a random drawing to show that the observed correlation is stronger than expected by chance. However, it seems to me that this control fails to capture the complex relationships between all the parameters involved. Most notably, because β_m and β_p values are positively correlated (r = 0.37) and because the $\Delta\beta_m$ between two paralogs is –on average- less than the $\Delta\beta_m$ between two randomly selected genes, the random drawing used by the authors is likely not an appropriate control.*

We do agree with the reviewer that our use of this correlation was misguided and that our assessment of its significance was not rigorous enough. Although we should have made it clearer in the original text, we saw this relationship between divergence ratio *D* and the log_2-fold change of estimated protein abundance (computed from the β_m and β_p rates) as a further illustration that transcriptional changes have driven expression divergence within paralog pairs (in addition to the original **Fig 1A**) and, consequently, as a minor addition to the paper. Because the dependent and independent variables are both obtained from β_m and β_p, such a positive correlation is expected simply from the fact that the β_m fold-changes among paralog pairs have greater variance than the β_p ones, which was already shown in **Fig 1A**. As such, this relationship adds very little new information on the expression divergence patterns of yeast duplicates. This is clear from our simulation results (**Figs 4-5** of the original manuscript), where the replication of the aforementioned correlation most often coincides with the obtention of realistic magnitudes of divergence in both β_m and β_p (as shown by low KS statistics and p-values > 0.05 for Mood's median test, respectively in **Figs 4A** and **5A**). Since this correlation is mostly redundant with our main result and taking it into consideration or not has very little influence over our conclusions,

we decided to remove it entirely from the paper. This helps make our manuscript more focused and avoids confusion introduced by this misguided addition. The only mention of divergence ratio *D* which subsists in the new version of our article is in **Fig S5**, where it is strictly used to measure how strongly the expression divergence within a paralog pair is biased toward transcriptional changes.

3. *In the discussion, the authors state that: "The current distribution of transcription and translation rates among yeast genes has previously been attributed to the optimization of the cost-precision trade-off [14]. Accordingly, suggesting that such constraints are not necessarily involved in the divergence of paralogs and that a difference of mutational target sizes could suffice may appear contradictory." (page 13, line 492) One possible solution to this apparent contradiction is that the cost-precision model proposed by Hausser et al (2019) explains the large-scale trends observed in bacteria, yeast and humans (why there are (almost) no genes with high transcription rate and low translation rate) while the divergence in expression between paralogs focuses on a narrower scale of variation (the average difference in β_m between paralogs is less than 10-fold while the patterns observed by Hausser emerge when looking at β_m values spanning 5 orders of magnitude). When looking at relatively small changes in expression level, the mutational effects might have a more important contribution than selection.*

We thank the reviewer for this very relevant comment. Our intention with this paragraph was to quickly mention that our simulated paralogous genes very rarely exceed the boundary described by Hausser *et al.* (2019), even when the constraints used to explain it are completely removed from the modelling framework. Not providing any potential explanation however likely made our comment less clear and enhanced the contradiction with (Hausser et al., 2019), instead of diminishing it. Because it seems like the most logical explanation, we have added the reviewer's hypothesis and the corresponding paragraph of our discussion now reads as: *"Since the current distribution of transcription and translation rates among S. cerevisiae genes has previously been attributed to the optimization of the precision-economy trade-off (Hausser et al. 2019), suggesting that such constraints may not be needed to explain the divergence patterns of paralogs might appear contradictory. This is especially true considering the significant energetic costs of even small increases of transcription and translation (Wagner 2005; Lynch and Marinov 2015). Yet, even when precision-economy considerations are fully neglected under the minimal model, extended in silico evolution results in only minor deviations from the reported distribution of genes in the transcription-translation space (Fig S14). One plausible explanation could be that precision-economy constraints impact evolutionary trajectories on longer timescales and/or along greater ranges of variation, while mutational effects dominate on the shorter timescales and smaller expression changes associated to the divergence of duplicates"*.

4. *One argument in favor of the mutational target sizes model is that the pattern observed here is highly consistent between SSD and WGD-derived paralogs. Because it has been repeatedly demonstrated that these two types of paralogs tend to evolve under divergent types of selective pressures (see for example https://www.ncbi.nlm.nih.gov/pmc/articles/PMC3536658/ and https://www.ncbi.nlm.nih.gov/pmc/articles/PMC2246283/), it seems to me that a mutational model would make sense to explain a pattern shared by SSD and WGD.*

As pointed out by the reviewer, the similarity between the divergence patterns of WGD- and SSD-derived paralogs is a surprising observation, particularly considering that it also holds in plants between tandem duplicates and WGD-derived ones (Wang and Chen, 2019). This may support the arguably simpler and more general mutational model, as suggested above. Accordingly, we have added the following to the fourth paragraph of our discussion, where we discuss that a mutational target size difference emerges as the preferred hypothesis: "*The high similarity between the expression divergence patterns of WGD- and SSD-derived paralogs (Figs 1 and S1; (Wang and Chen 2019)), which have been shown to differ substantially in their initial properties and subsequent evolutionary trajectories (Hakes et al. 2007; Guan et al. 2007; Fares et al. 2013), may also support such a more general mutational mechanism*".

Reviewer 2:

1. *The transcription and translation rates used by the authors were taken from a study (Hausser et al) that ignored the roles of mRNA and protein turnover. This is a major problem, and could be partly addressed, at least on the mRNA side, by using actual transcription and mRNA decay rates calculated by 4-thioU incorporation studies (e.g. RATE-seq from the Gresham lab, and many others). The translation rates are a bit trickier. In yeast, it has been established that translation and mRNA decay are coupled processes (e.g. Presnyak et al., Cell), such that slow translation and/or ribosome collision is positively correlated to mRNA decay. This complicates attempts to model the processes independently, as increases in ribosome loading (an increase in translation efficiency) could lead to reduced mRNA levels in genes with suboptimal codons. Indeed, Shen et al., (2022) showed synonymous mutations are often non-neutral. This creates additional complications as mutations to more common codons may increase expression of the protein while reducing the apparent translation rate from Ribo-seq studies (ribosomes complete translation more rapidly, so steady-state occupancy decreases). This increase in translation efficiency would "look" like an increase in transcription because better translation results in more stable mRNAs. My general concern is that the transcription and translation rates used are likely not accurate and the coupling of translation to decay complicates the issue beyond the approach taken by the authors.*

We thank the reviewer for raising this issue. Completely neglecting gene-to-gene variation in decay rates was indeed an oversight on our part, largely because this consideration is mostly absent from studies on the evolution of gene expression and because we thought that considering both transcription and translation was already quite complex and that there was insufficient data on how

decay rates evolve to consider this aspect. We now realize that we need to treat this point in the manuscript.

To address this, we focused on validating that our initial observation – that yeast paralogs mostly diverged at the transcriptional level – is not a product of overly simplistic assumptions made in the calculation of transcription and translation rates. Modeling the evolution of mRNA and protein decay rates appears to be out of the scope of this work, especially considering that opposite changes at the levels of synthesis and decay (e.g. increase in transcription vs decrease in mRNA decay) are entirely equivalent within our minimal model of post-duplication evolution. As such, the evolution of decay rates was already included implicitly in most of our simulations.

To validate the transcription rates β_m used, we followed the reviewer's suggestion and recalculated them using experimental measurements of transcript turnover. The original calculations from Hausser et al. (2019) were made under the assumption that mRNA decay is constant across genes. Since the experimental data used (mRNA abundance) reflects the combined effect of synthesis and decay, this assumption may have had a major impact on the obtained transcription rates, as pointed out by the reviewer. We used 4 datasets obtained using different experimental approaches, including the aforementioned RATE-seq (Eser et al., 2014; Geisberg et al., 2014; Munchel et al., 2011; Neymotin et al., 2014). In all cases, our initial observations were confirmed, as the relative divergence in transcription remained significantly higher than the relative translational divergence, while the two correlations remained qualitatively identical (**Figs 1 and S1**). We also went a step further and generated simulated data to ensure that, even in conjunction with experimental noise, the assumption of constant mRNA decay rates could not have falsely made relative transcriptional changes appear greater than translational divergence (**Fig S3**).

To validate the translation rates β_p, we did not focus on variations in protein decay rates and instead directed our attention towards confirming that the abundance of ribosomal footprints is representative of the translational flux. While we wrote in the previous version of our manuscript that Hausser et al. (2019) obtained the β_p rates under the assumption that protein decay is constant across yeast genes, this was not the most accurate description of their approach. Rather, their true supposition is better described as an assumption of constant translational elongation rates across all transcripts. Hausser et al. (2019) indeed used the median protein decay rate α_p to estimate the total translational flux in a yeast cell, from which they assigned translation rates β_p to each gene according to the abundance of their ribosomal footprints (reproduced as **Eq. 3** in our paper). This is obviously a simplification, and the reviewer is right to point out that such Ribo-seq measurements might not reflect the full complexity of translational variations. We are nonetheless confident that the corresponding translation rates are representative. It has been shown that ribosomal footprints abundance from Weinberg et al. (2016), which have been used by Hausser et al. (2019), are very well correlated with total protein synthesis in yeast ($r = 0.81$ and $\rho = 0.83$), as measured by pulsed stable isotope labeling of amino acids in culture (Riba et al., 2019). Moreover, changes to the elongation rate are not the major driver of translational variations between yeast genes, covering a ~20-fold range compared to ~100-fold for initiation rate variations (Riba et al., 2019; Shah et al., 2013), and are often correlated with variations in initiation (Riba et al., 2019). As such, cases where slower/faster elongation markedly increases/decreases steady-state ribosomal occupancy and skews Ribo-seq measurements of translation must be rather rare. We

still performed additional analyses to validate the accuracy of the translation rate $\log_2$-fold changes we obtained using the β_p values from Hausser et al. (2019). For all paralog pairs for which data was available, we computed protein abundance $\log_2$-fold changes from the combination of mRNA abundances, translation rates (Hausser et al., 2019) and experimental measurements of protein turnover (Christiano et al., 2014; Martin-Perez and Villén, 2017) using **Eq. 5**. We then compared them to experimentally measured protein abundance differences (Ho et al., 2018; Martin-Perez and Villén, 2017; Wang et al., 2015). If the β_p were accurate, their combination with mRNA abundances and protein decay measurements – whose validity is less ambiguous – would replicate observed differences in protein abundance. This revealed a very good correlation (**Fig S1E**), which suggests that β_p values (and more importantly the corresponding fold changes) are representative of true translational output, and thus adequate for the current work.

An alternative to the use of Ribo-seq measurements could have been to infer translation rates from experimental measurements of protein abundance and protein turnover rate, assuming that their product is equal to the total synthesis flux. Under this approach, β_p rates would however not be related to any direct measurement of translation. More importantly, transcription and translation rates would result from distinct experiments using widely different approaches, and thus be less easily comparable. Comparisons of the magnitudes of transcriptional and translational fold changes across paralog pairs would indeed be more likely to be dominated by noise. Within the scope of our work, the biggest advantage associated with the β_m and β_p values from Hausser et al. (2019) is that they have been inferred from the same experiment – paired mRNA-seq and ribosome profiling – and are thereby directly comparable. As such, while the abundance of ribosome footprints is not a perfect measurement of translation, we think that the β_p reported by Hausser et al. (2019) are the most adequate translational measurement to investigate the joint evolution of transcription and translation within paralog pairs.

Finally, the reviewer is right to point out that modeling the evolution of transcription and translation independently may be overly simplistic. We however note that our additional simulations using correlated mutational effects (**Fig 5** in both our original and new manuscript) do account, although obviously not entirely, for potential evolutionary couplings between the two processes. We have now made this clearer in the text, while also explaining that any coupling between translation and mRNA decay can also be accounted for by such mutational correlations, at least when considering our minimal model. Within this model, only the respective impacts of transcriptional and translational changes on steady-state mRNA and protein abundances matter, such that an increase in mRNA decay is entirely equivalent to a decrease of transcription. Correlations between the transcriptional and translational effects of mutations can thus model the likely coupling between mRNA decay and translation, especially since the empirical divergence patterns to which we compared the results of our simulations have been obtained under the assumption of constant mRNA decay – such that transcriptional changes compound variations in decay. A caveat is however that this is not entirely true in the precision-economy version of our model, as changes to mRNA decay and transcription do not have the same effects on expression noise and cost. The second paragraph of the last subsection of our *Results* (*Revisiting the hypotheses when considering transcription-translation couplings and biased mutational effects distributions*) now begins with: "*Many mutations in the transcribed region of a gene may for instance simultaneously have transcriptional and translational effects, as the identity of the translated codons might*

affect both mRNA stability and translation (Presnyak et al. 2015; Chen et al. 2017; Chan et al. 2018)". We have also added the following to the third paragraph of the same subsection: "*This second addition to the model, which allows mutations to act on both m and p at once, can potentially account for regulatory responses as well as for the coupling between mRNA decay and translation efficiency. Within our minimal model, under which identical changes to transcription and mRNA stability are entirely equivalent (as only their effect on protein abundance matters), correlated effects on β_m and β_p can indeed represent such a coupling*". We note that we have not made any assumptions about the synonymous or nonsynonymous nature of mutations: we simply model their effects on expression level.

2. *The modeling section assumes the duplicated genes were only under selection to maintain current protein levels. Since the WGD event, when presumably the entire genome was duplicated, Saccharomyces yeast have gone through a "pruning" process, such that the remaining ohnologs ~ 100 M years later most likely include many genes whose doubling was either completely neutral or offered some advantage. This is relevant to the parabolic fitness curves used for modeling, which assume maintaining steady-state protein is always most-fit. This assumption is at odds with Keren et al., 2016 Cell), who found a variety of fitness curves for 81 yeast genes, that also depend on growth conditions.*

We had not provided sufficient justification for our minimal model of post-duplication evolution in our original manuscript, and we thank the reviewer for this opportunity to correct this. We now provide such explanations in the fourth paragraph of the subsection *A minimal model of post-duplication expression evolution* of our *Results*. In addition to the evidence which supports it, our choice of this framework was based on its general applicability to any paralog pair and its simplicity, which made it a good starting point. There is no doubt that more complex models could be considered in the future.

By using absolute dosage subfunctionalization (or quantitative subfunctionalization) as the foundation of our minimal model, we have not assumed that duplicated genes have only been under selection to maintain optimal (cumulative) protein abundance levels. Rather, we have assumed that this mechanism has played an important role in the evolution of paralog pairs and chose to focus on this aspect of their divergence. The interest of our simulations lies in part in the fact that we show that this simple process can generate realistic evolutionary patterns in transcription and in translation. There is evidence that quantitative subfunctionalization has shaped the evolution of most WGD-derived paralogs, as the model was first proposed based on genome-wide trends in yeast and *Paramecium* (Gout and Lynch, 2015). Even in cases where other mechanisms – such as neofunctionalization – are currently opposing the loss of ancient duplicates, it is likely that quantitative subfunctionalization has been involved earlier in evolution. The evolutionary trajectories of neofunctionalized duplicate sodium channels in teleost fish have for instance been shown to be consistent with the latter model (Thompson et al., 2016). In yeast, there is even strong evidence that selection to maintain cumulative expression may still be one of the main factors opposing the loss of at least some paralogs. This is exemplified by the many duplicate pairs which display synthetic lethality, as well as by instances where the deletion of one paralog results in compensatory upregulation of its duplicate (DeLuna et al., 2010). Keren et al. (2016) in addition provided a striking example of such still-impactful quantitative subfunctionalization, by showing

that fitness depends on the cumulative expression of paralogs *TUB*1 and *TUB3* rather than on any of their individual expression levels.

The reviewer is right to point out the importance of the gene loss which occurred following the yeast WGD event. Because loss-of-function mutations are presumably very frequent, genes whose duplication was deleterious or neutral most likely reverted to the single-copy state (Lynch and Force, 2000). To account for this process, we made sure to select only paralog pairs for which the loss of a duplicate immediately after the duplication event is deleterious (initialization of the simulations, in **Methods**). Previous work assumed that, in the case of a WGD, the immediate post-duplication expression levels are perfectly optimal (Gout and Lynch, 2015). For our model to be more general, we relaxed this assumption and instead postulated that the expression doubling would overshoot the protein abundance optimum, while still being fitter than the return to a single-copy state. As such, we have not assumed that maintaining current (initial) expression level is always optimal, but rather that an optimum exists, which is supported by experiments. This is now highlighted in the section *A minimal model of post-duplication expression evolution*, paragraph 6 (lines 268-278).

The "pruning" of duplicates was likely not limited to pairs which could revert immediately after the duplication event. With increasing expression divergence within gene pairs, the loss of the least expressed copy would gradually become neutral in more of them, leading to further loss of paralogs (Gout and Lynch, 2015; Thompson et al., 2016). A limitation of our work is therefore that we did not include any gene loss during the simulations – as mentioned in the discussion –, and we thank the reviewer for insisting on this point. We tried implementing loss-of-function mutations within our framework, so that gene loss would occur when tolerated by selection (neutral or beneficial), but this made it impossible for the end condition of the simulation to be attained. Instead, we chose to validate our results by performing all tolerated loss-of-function mutations at the end of simulations, before the calculation of summary statistics. As shown in **Fig S15**, this revealed results qualitatively identical to those we report in the main *Results*, although the fit to empirical divergence patterns was slightly reduced. While the absence of gene loss remains a limitation of our approach, this additional analysis strengthens our conclusions. This is now included in the last paragraph of our *Discussion* (lines 735-741). Finally, we also note that the patterns of divergence we observed are based on pairs which have been maintained as duplicates until now. Our simulations, which do not allow for gene loss, therefore represent the evolution of gene pairs such as those present in the dataset we based our observations on – provided that our randomly generated paralog pairs are representative of such genes. While an imperfect approximation, the minimal fitness function curvature threshold that we enforced ensures that the simulated pairs share at least some properties with the paralogs which have been maintained in yeast.

Using a strictly concave fitness function, as in Dekel & Alon (2005) and Gout et al. (2010), appeared to be the most realistic and general way to connect protein abundance to fitness in our study. We are aware however that, according to Keren et al. (2016), expression-fitness relationships can have a wide variety of shapes across a selected set of yeast genes. Taking into account all this diversity would nonetheless be outside the scope of this work, where a general model of the expression divergence of paralogous genes was desired. We note that in the case of

this specific study, the actual expression of the genes for which fitness was estimated was not measured. It was inferred from the fusions of various promoters with a fluorescent protein. It is also important to note that more recent and comprehensive experimental work reported less varied fitness function shapes, in yeast (Arita et al., 2021) as well as in bacteria (Hawkins et al., 2020).

Additionally, the evolutionary relevance of such fitness function measurements is unclear. The fitness differences which are visible to selection in large microbial populations are several orders of magnitudes smaller than what can be measured experimentally. As such, fitness has likely been assayed over a wider range of expression levels than accessible evolutionarily, and the shape of the corresponding function in the relevant interval may be different when viewed at a high enough resolution. Because of this uncertainty, we preferred to consider only strictly concave fitness functions (with one unique maximum) – an idealized but widely applicable scenario. If fitness is related to the abundance of a protein, there must intuitively be an expression level which is most fit. Fitness can also be expected to decrease gradually away from this optimum. At low expression, the protein may not be abundant enough to carry out its cellular function, while metabolic cost would increase with expression and high abundance might have other deleterious consequences (such as aggregation). Accordingly with this intuition, papers exploring how selection acts on expression levels frequently assume simple concave fitness functions (Dekel and Alon, 2005; Gout et al., 2010; Thompson et al., 2016), as we did. Whether the regions of reduced fitness are accessible experimentally may vary between genes according to the curvature of their fitness function, further reconciling this assumption with observations. In the current work, we have considered a wide range of curvatures, including low values resulting in an almost flat fitness function. While these expression-fitness relationships can certainly vary across growth conditions, considering the impact of environmental fluctuations was out of the scope of this work.

3. *The modeling section also assumes that selection acts only on expression level and not on noise (as far as I can tell). This is almost certainly incorrect and ignores trade-offs on fitness costs commonly seen in yeast (e.g. Gavin Sherlock's work). Indeed gene duplication most likely decreases expression noise, which could be beneficial depending on the function of each gene (e.g. housekeeping genes vs stress response genes). I am not sure how the authors could address this, but given that the genes studied are the only ones to survive WGD, they are an unusual set compared to denovo gene duplications.*

We have explicitly included expression noise within our precision-economy (formerly cost-precision) model, which investigates how the trade-off between minimizing noise and transcriptional cost may impact the divergence of duplicates. We have made sure to make this clearer and we now write *"First, expression noise (and thus the importance of precision) is explicitly taken into account by considering the mean fitness of a population of cells expressing two paralogs at a mean cumulative protein abundance P_{tot} with standard deviation σ_{tot}, which itself depends on the relative contribution of transcription to overall expression (Hausser et al. 2019) (**Fig 2B III**; **Methods**)"* in the fifth paragraph of the subsection *A minimal model of post-duplication expression evolution* of the *Results*. It is true that we did not include selection on the level of noise itself. Rather, it is the impact of noise on mean protein abundance, and thus on population fitness, which is visible to selection. We note that, this way, it is always beneficial to reduce noise within our framework.

This benefit may however be very small if the curvature of the fitness function is low, and is offset at some point by the increase in transcription cost that is necessary to decrease noise (the basis of the precision-economy trade-off). Direct selection on noise level would likely only be appropriate when a higher noise would be favored, for instance for stress response genes or in the face of environmental fluctuations. Modeling such presumably rarer cases was however out of the scope of this work.

Whether gene duplication inherently reduces noise is not entirely clear. It has been suggested that, for a given protein abundance, two gene copies may be associated with less fluctuations than a single one and that this may favor the fixation of paralogs (Rodrigo and Fares, 2018). This is nevertheless not the case within our framework, because the equation we used (see **Extended Methods**) accounts for the scaling with protein abundance of variance due to intrinsic noise, which causes less expressed genes to be relatively noisier (Bar-Even et al., 2006). Because of this relationship, expressing a protein at the same total level from one singleton or from two duplicates results in the same variance within our model. As this scaling has been explained by the Poissonian nature of gene expression (Bar-Even et al., 2006; Hausser et al., 2019; Swain et al., 2002) – rather than through an evolved correlation –, it appears adequate to consider it when assessing how mutations and gene duplication events affect noise. We note however that the doubling of expression associated with gene doubling does reduce noise (as measured by the coefficient of variation on cumulative protein abundance) in our precision-economy model, especially considering that this occurs entirely transcriptionally. If duplicated genes were inherently less noisy and this presented an advantage, our results and conclusions would likely not be affected. Such a beneficial effect of gene duplication would indeed matter when studying the fixation of paralogs, while we were instead interested in the expression divergence of gene pairs which have already reached fixation.

Finally, the reviewer is right to point out that the duplicates remaining from the yeast WGD event are an unusual set of genes. This is the reason why we enforced a threshold of minimal fitness function curvature when generating paralog pairs in our simulations. This way, we selected a set of genes which could reasonably be expected to be maintained as duplicates for an extended period of time. A limitation of this approach is however that we generated paralog pairs from the complete yeast genome, while it's possible that no current yeast gene is representative of pre-WGD ancestral genes. This is especially true considering that all current singletons in yeast were also initially duplicated at the time of the WGD. We are nonetheless confident that randomly sampling ancestral singletons among current yeast genes – and filtering them – was the best way to generate a large number of duplicate pairs for our simulations. Any inference of the transcription and translation rates of the true ancestors of present-day paralogs would have been based on the divergence patterns which we wanted to study, and would thus have been circular. We now include this in the last paragraph of our *Discussion*: "*An alternative could have been to restrict our simulations to duplicate pairs which were destined to be retained for an extensive period. Using current transcription and translation rates of paralogs to infer the expression levels of ancestral singletons and then investigate the divergence of duplicates would however have proved circular"*. Moreover, since we focus on the relative divergence between paralogs, the absolute magnitude of the transcription and translation rates of the simulated genes probably have little influence over the results.

4. *The modeling section is difficult to follow due to its complexity. I am not sure how to fix this but, as a reader, it is very difficult to understand some of the sentences and figures. This is not due to improper grammer, but rather the complexity of the system and, perhaps, too much jargon. A reduction in jargon and reworking the figures could make the manuscript more accessible to a general readership, e.g. at PLoS Genetics, who otherwise have difficulty comprehending the methods and results. This is a big problem with the figures, which are almost entirely graphs and could benefit from more helpful illustrations to aid in interpretation. This would also be in the author's interest, as it would increase citations in the future if readers can better comprehend their work.*

We thank the reviewer for pointing out that the lack of schematics in the modeling section (outside of **Fig 2**) could make it more difficult for readers to understand our results. We have now added schematics to **Fig 4** and **Fig 5** (panel A in both cases), which summarize how the results presented in the other panels of the figures have been generated. We have also made sure to improve our explanations throughout the modeling section, both regarding the design of the model and the interpretation of the figures, so as to make our manuscript more accessible (detailed later in the document).

5. *The manuscript might be better suited for a more specialized journal (e.g. JEB or Journal of Theoretical Biology), whose readers would be more prepared for the heavy use of complex modeling.*

We believe that our manuscript is better suited for a journal with a general readership such as *PLOS Genetics*, because its interest lies more in the ideas that we put forward (for instance, that transcription might have a larger mutational target size than translation, the importance of studying both the transcriptional and translational components of expression divergence, the necessity to fully characterize the distributions of mutational effects as well as their biases and how the recently described precision-economy trade-off may uniquely affect duplicated genes) than in the modeling approach we used. We also think that our work identified very important biological parameters to be measured experimentally in the future, such that our results will be of interest to geneticists and experimentalists.

<u>Reviewer 3:</u>

1. *Equation 1: Move this equation to line 68, so that it is presented after the θ parameters are explained. The term folddupli does not seem to be used anywhere else in the manuscript, so why use that name here? In the rest of the manuscript it is just called Log2 fold-change.*

The reviewer is right that introducing a $fold_{dupli}$ term which was used nowhere else in the manuscript was a mistake. This has been replaced by "log_2-fold change" in the equation.

2. *Figure 2A, left: I have an intuitive understanding of what a "cost-precision trade-off" means, but I found the cartoons in Figure 2A more confusing than helpful and the legend didn't explain them. Are the words Precision and Cost on the two small graphs titles, or are they the quantities on the X-axis? Or is the X-axis Transcription on all 3 graphs?*

The x axis on each of the two small graphs should have been labelled "Transcription" as well, but these two labels had been removed in a last-minute correction to the figure. We thank the reviewer for pointing out this mistake. We have also modified our terminology throughout the paper (including in this figure) to use the expression "precision-economy trade-off". This should be much clearer, as precision and economy vary inversely with transcription rate, which emphasizes how this results in an evolutionary trade-off. We have also made sure to clarify our explanation of the precision-economy trade-off, in the second paragraph of the section *A minimal model of post-duplication expression evolution* of the *Results*.

3. *I was able to understand Figures 1-3, but I had a lot of difficulty with Figure 4A and the associated text on lines 284-315. The authors explain that the distances between the empirical and simulated distributions of 4 quantities (line 296) is summarized by a single number, the KS statistic. But I don't know how to read Figure 4A because I don't know whether a high or low value of the KS statistic indicates a good fit, i.e. low distances. Consequently, I don't understand this statement (line 300), referring to the N = 1 x 10^6 panel in Fig 4A: "When a high efficacy of selection is assumed – intuitively more likely for Saccharomyces yeasts –, only the minimal model can reasonably replicate the empirical patterns of divergence.". I can see that the minimal model has more variation in the KS statistic than the cost-precision model, but how am I supposed to know if a particular KS value does (or does not) reasonably replicate the empirical patterns of divergence? The other panels of Fig 4 are easier to read because the 95% confidence intervals are shown.*

It is true that we had not sufficiently explained the results presented in **Figs 4-5**. We thank the reviewer for this opportunity to improve our manuscript. In addition to adding schematics to each figure, we have also added a paragraph explaining how the results have been generated in each case (lines 406-422 and lines 544-554).

We have made sure to explicitly mention which values of KS statistics are associated with a better fit. We now write "*The distance between the simulated and empirical distributions of relative divergence (log_2-fold change) in transcription and translation rates as well as protein abundance was first quantified using the Kolmogorov-Smirnov (KS) statistic. For each replicate simulation, the three resulting measurements were combined into a mean KS statistic, between 0 and 1, for which a lower value indicates a better overall fit*".

We have also clarified the statement which the reviewer rightfully found difficult to understand. What we meant is that the minimal model is the only one for which some of the tested mutational target size ratios are associated with low KS statistics. We agree that the use of "can reasonably replicate" was confusing, as there is no precise threshold of KS statistics for which the replication of the empirical distributions can be deemed adequate/reasonable. The much lower values which

can be reached for the minimal model however show that it performs better than the alternative. The improved explanation is: " *When a high efficacy of selection is assumed, the minimal model is by far the most accurate, as shown by the attainment of much lower mean KS statistics (as low as ~0.07, compared to values >0.2 for the other model). The best fit is obtained when a higher probability of mutations affecting β_m is assumed, and especially when $\frac{P_{\beta m}}{P_{\beta p}}$ is between 3 and 6, which supports the hypothesis of a larger mutational target size for transcription*".

4. *Similarly, I don't see how to extract the conclusion "Interestingly, the best agreement with the real patterns of evolution is obtained for a relatively modest difference of mutation probabilities" (line 313) from Figure 4A. Readers need to be given some guidance about how to interpret this Figure. What are we looking for?*

We have removed this sentence, as it was confusing. We however clarified the last sentence of this section of the *Results* (*A difference of mutational target sizes may better explain the observed divergence patterns*), which repeated the same idea. It is now written as: "*Although the relative mutational target sizes of transcription and translation regulation are not known, the fact that the best agreement with our observations (lowest mean KS statistic) is obtained for a modest difference of relative mutation probability means that the bias need not be important to impact evolution*".

5. *Also in Figure 4A (and several other figures), the X-axis is labelled "Relative mutational target sizes (βm/βp)". Shouldn't this be Pβm/Pβp , because the β values are rates (line 58) and the Pβ values are relative mutational target sizes (line 187 and 271) ?*

The reviewer is right that these axis labels introduce unnecessary confusion. They have been replaced by "$\left(P_{\beta m}/P_{\beta p}\right)$" in all figures.

<u>Other corrections and improvements</u>

- During the revision process, we noticed a mistake in our code, in which **Eq. 15** computing fixation probabilities was written using log_{10} instead of the natural logarithm. For this reason, we had to repeat the entirety of our simulations. To speed up the process, we used a computing cluster and decided to slightly increase the number of simulated paralog pairs in each run to 2500. Our conclusions globally remained the same, but this correction had an effect on the results presented in **Fig 5**. Contrary to what we reported in the previous version of our manuscript, no combination of parameters can now simultaneously replicate all the features of the empirical divergence of yeast paralogs. The addition of biases (skewness and correlations) to the distributions of mutational effects however still has a strong effect on the replication of the divergence correlations. As such, these results still show that the expression divergence patterns could plausibly be fully replicated by at least one of our models.

- We have also noticed a mistake in **Fig 1D** (Fig 1C in our original submission), which we have now corrected. The signed fold changes had been scaled in log_{10} instead of log_2, which caused the range of values to be much smaller than for the absolute fold changes shown on other panels (**Fig 1 A-C** in the current version). This mistake had no impact on our results or conclusions, as the signed log_2-fold changes were only used to compute the Spearman correlation, which is based on ranks and is thus unaffected by the units or log-scaling of the values used.

- We realized that the way we had initially framed our research question made it seem as if the relevance of our modeling work depends entirely on the validity of our initial observation. Our explorations of mechanisms which could favor transcriptional evolution is however interesting regardless. We thus reworked our introduction to mention the many previously published observations which suggest that the evolution of gene expression levels occurs mostly through transcriptional changes. In this context, yeast paralog pairs become a model system which can be easily studied to better understand how such a difference in evolutionary rates could emerge.

- In order to better contextualize the evolutionary correlations that we highlight between the transcriptional and translational changes within paralog pairs (**Fig 1 C-D**), we added a supplementary figure (**S4 Fig**) showing the spurious correlations which would be expected from the fact that mRNA abundance m is used in the calculation of β_m and β_p. These expected relationships are distinct from what we show in **Fig 1 C-D** – especially in the case of the signed log_2-fold changes –, which suggests that these correlations are meaningful and significant.

- Although reviewers did not comment specifically on this section, we have also clarified our explanation of how the precision-economy trade-off by itself favors transcriptional divergence within paralog pairs (subsection *The precision-economy trade-off is sufficient to promote transcriptional divergence*). The following was added at the end of the penultimate paragraph of the subsection (lines 335-340): "*This occurs due to interactions between transcription- and translation-acting mutations introduced by the precision-economy trade-off. Further transcriptional changes can for instance be expected to be favored by selection after the fixation of a mutation altering β_m, as they have the potential to compensate effects on both precision and economy, while a change of translation can individually only act on precision*".

- We have overhauled our discussion, both to clarify it and to adjust it to the new framing of the research question.
  - We have added a paragraph (sixth one, lines 639-656) discussing whether the two mechanisms that we have studied may apply widely to all genes – and thus might explain general trends of faster transcriptional evolution – or are instead likely to be specific to duplicated genes.
  - The paragraph speculating on the wider evolutionary consequences of the mostly transcriptional divergence of paralogs (eighth one, lines 673-691) has been

reworked, both to make its speculative nature clearer and to clarify it. It did not make sense to oppose the resolution of noise-control conflicts to regulatory neofunctionalization, as we did previously.
  - The paragraph discussing the fact that we ignored functional changes within proteins as well as how the precision-economy trade-off may affect the neofunctionalization paths available during the process of quantitative subfunctionalization has been split into two. The first part (now lines 692-703) discusses whether it was reasonable to focus only on expression divergence within paralog pairs. The second (now lines 704-714) describes how early expression changes, if they were dictated by the precision-economy trade-off, could shape the later evolutionary trajectories of duplicated genes.

- The abstract and the conclusion have both also been modified, to ensure that they are consistent with the new framing of the work, as well as with the slightly modified results.

- In order to shorten the *Methods* a bit, we have moved some details related to the fitness functions, the selection of the post-duplication change in optimal (cumulative) protein abundance, the estimation of expression noise and the selection of valid ancestral singletons to *Extended Methods* in the supplementary material. Throughout, we have also shortened the *Methods* and vastly reduced the repetition of information which was already mentioned in the *Results* and/or in the figure legends.

Cited works:

Arita, Y., Kim, G., Li, Z., Friesen, H., Turco, G., Wang, R.Y., Climie, D., Usaj, M., Hotz, M., Stoops, E.H., Baryshnikova, A., Boone, C., Botstein, D., Andrews, B.J., McIsaac, R.S., 2021. A genome-scale yeast library with inducible expression of individual genes. Mol. Syst. Biol. 17, e10207. https://doi.org/10.15252/msb.202110207

Bar-Even, A., Paulsson, J., Maheshri, N., Carmi, M., O'Shea, E., Pilpel, Y., Barkai, N., 2006. Noise in protein expression scales with natural protein abundance. Nat. Genet. 38, 636–643. https://doi.org/10.1038/ng1807

Belle, A., Tanay, A., Bitincka, L., Shamir, R., O'Shea, E.K., 2006. Quantification of protein half-lives in the budding yeast proteome. Proc. Natl. Acad. Sci. 103, 13004–13009. https://doi.org/10.1073/pnas.0605420103

Christiano, R., Nagaraj, N., Fröhlich, F., Walther, T.C., 2014. Global Proteome Turnover Analyses of the Yeasts S. cerevisiae and S. pombe. Cell Rep. 9, 1959–1965. https://doi.org/10.1016/j.celrep.2014.10.065

Dekel, E., Alon, U., 2005. Optimality and evolutionary tuning of the expression level of a protein. Nature 436, 588–592. https://doi.org/10.1038/nature03842

DeLuna, A., Springer, M., Kirschner, M.W., Kishony, R., 2010. Need-Based Up-Regulation of Protein Levels in Response to Deletion of Their Duplicate Genes. PLOS Biol. 8, e1000347. https://doi.org/10.1371/journal.pbio.1000347

Eser, P., Demel, C., Maier, K.C., Schwalb, B., Pirkl, N., Martin, D.E., Cramer, P., Tresch, A., 2014. Periodic mRNA synthesis and degradation co-operate during cell cycle gene expression. Mol. Syst. Biol. 10, 717. https://doi.org/10.1002/msb.134886

Geisberg, J.V., Moqtaderi, Z., Fan, X., Ozsolak, F., Struhl, K., 2014. Global Analysis of mRNA Isoform Half-Lives Reveals Stabilizing and Destabilizing Elements in Yeast. Cell 156, 812–824. https://doi.org/10.1016/j.cell.2013.12.026

Gout, J.-F., Kahn, D., Duret, L., Paramecium Post-Genomics Consortium, 2010. The relationship among gene expression, the evolution of gene dosage, and the rate of protein evolution. PLoS Genet. 6, e1000944. https://doi.org/10.1371/journal.pgen.1000944

Gout, J.-F., Lynch, M., 2015. Maintenance and Loss of Duplicated Genes by Dosage Subfunctionalization. Mol. Biol. Evol. 32, 2141–2148. https://doi.org/10.1093/molbev/msv095

Hausser, J., Mayo, A., Keren, L., Alon, U., 2019. Central dogma rates and the trade-off between precision and economy in gene expression. Nat. Commun. 10, 68. https://doi.org/10.1038/s41467-018-07391-8

Hawkins, J.S., Silvis, M.R., Koo, B.-M., Peters, J.M., Osadnik, H., Jost, M., Hearne, C.C., Weissman, J.S., Todor, H., Gross, C.A., 2020. Mismatch-CRISPRi Reveals the Co-varying Expression-Fitness Relationships of Essential Genes in Escherichia coli and Bacillus subtilis. Cell Syst. 11, 523-535.e9. https://doi.org/10.1016/j.cels.2020.09.009

Ho, B., Baryshnikova, A., Brown, G.W., 2018. Unification of Protein Abundance Datasets Yields a Quantitative Saccharomyces cerevisiae Proteome. Cell Syst. 6, 192-205.e3. https://doi.org/10.1016/j.cels.2017.12.004

Johri, P., Gout, J.-F., Doak, T.G., Lynch, M., 2022. A Population-Genetic Lens into the Process of Gene Loss Following Whole-Genome Duplication. Mol. Biol. Evol. 39, msac118. https://doi.org/10.1093/molbev/msac118

Lynch, M., Force, A., 2000. The probability of duplicate gene preservation by subfunctionalization. Genetics 154, 459–473. https://doi.org/10.1093/genetics/154.1.459

Martin-Perez, M., Villén, J., 2017. Determinants and Regulation of Protein Turnover in Yeast. Cell Syst. 5, 283-294.e5. https://doi.org/10.1016/j.cels.2017.08.008

Munchel, S.E., Shultzaberger, R.K., Takizawa, N., Weis, K., 2011. Dynamic profiling of mRNA turnover reveals gene-specific and system-wide regulation of mRNA decay. Mol. Biol. Cell 22, 2787–2795. https://doi.org/10.1091/mbc.E11-01-0028

Neymotin, B., Athanasiadou, R., Gresham, D., 2014. Determination of in vivo RNA kinetics using RATE-seq. RNA N. Y. N 20, 1645–1652. https://doi.org/10.1261/rna.045104.114

Riba, A., Di Nanni, N., Mittal, N., Arhné, E., Schmidt, A., Zavolan, M., 2019. Protein synthesis rates and ribosome occupancies reveal determinants of translation elongation rates. Proc. Natl. Acad. Sci. 116, 15023–15032. https://doi.org/10.1073/pnas.1817299116

Rodrigo, G., Fares, M.A., 2018. Intrinsic adaptive value and early fate of gene duplication revealed by a bottom-up approach. eLife 7, e29739. https://doi.org/10.7554/eLife.29739

Shah, P., Ding, Y., Niemczyk, M., Kudla, G., Plotkin, J.B., 2013. Rate-Limiting Steps in Yeast Protein Translation. Cell 153, 1589–1601. https://doi.org/10.1016/j.cell.2013.05.049

Swain, P.S., Elowitz, M.B., Siggia, E.D., 2002. Intrinsic and extrinsic contributions to stochasticity in gene expression. Proc. Natl. Acad. Sci. 99, 12795–12800. https://doi.org/10.1073/pnas.162041399

Thompson, A., Zakon, H.H., Kirkpatrick, M., 2016. Compensatory Drift and the Evolutionary Dynamics of Dosage-Sensitive Duplicate Genes. Genetics 202, 765–774. https://doi.org/10.1534/genetics.115.178137

Wang, M., Herrmann, C.J., Simonovic, M., Szklarczyk, D., von Mering, C., 2015. Version 4.0 of PaxDb: Protein abundance data, integrated across model organisms, tissues, and cell-lines. Proteomics 15, 3163–3168. https://doi.org/10.1002/pmic.201400441

Wang, S., Chen, Y., 2019. Fine-Tuning the Expression of Duplicate Genes by Translational Regulation in Arabidopsis and Maize. Front. Plant Sci. 10, 534. https://doi.org/10.3389/fpls.2019.00534
