# Supplementary material for: Evolutionary trade-off and mutational bias could favor transcriptional over translational divergence within paralog pairs
Source: PLoS Genet. 2023 May 26;19(5):e1010756. doi: 10.1371/journal.pgen.1010756 (PMC10275480; doi:10.1371/journal.pgen.1010756)
Supplement: Attachment — Submitted filename: Response_reviews.pdf [file pgen.1010756.s019.pdf]

# <u>Response to reviewer comments</u>

<u>Reviewer 2:</u>

1. *My only comment is about the cartoons in Figure 2A (left, top). For clarity, shouldn't the Y-axes of the 2 small graphs be labeled "Precision" and "Economy" rather than "Fitness"? (Fitness is not mentioned in the text on lines 179-192 or the legend to Fig 2A).*

The reviewer is right that the relationship between fitness and both precision and economy could be made more obvious, such that the left part of Fig 2A would be easier to understand. We thank them for raising this issue. We now write explicitly in the accompanying text that maximizing the precision and economy of gene expression would provide fitness benefits: *"Accordingly, the precision and economy of gene expression cannot be maximized simultaneously, although it would provide fitness benefits, because they inversely depend on the relative contributions of transcription and translation"* (lines 174-176).

<u>Reviewer 3:</u>

1. *Minor comment - I'm not sure how PLoS journals handle images made on Biorender, and if you would be better off acknowleding that in the acknowledgement section than in figure legends.*

We have seen it acknowledged this way in other PLOS Genetics papers, so we have not made any change. We however thank the reviewer for pointing this out.

<u>General comments:</u>

All three reviewers agreed that the Results section contained too many methodological details and was challenging to read for this reason.

Reviewer 1: *"I also agree with reviewer #2 that some sections of the paper (especially the modeling part) are sometime difficult to follow, but I don't really have any solution to offer. It seems to me that the complexity is just inherent to the question being studied and the approach (complex simulations)."*

Reviewer 2: *"The resubmitted version of Aubé et al's manuscript is somewhat clearer and easier to follow than the original submission, though I still find some parts of it rather tough going. I can see that the authors made some effort to make the manuscript more accessible to generalist readers, but perhaps they could go further still."*

Reviewer 3: *"My one remaining concern is that the manuscript is very challenging to read, and should be made more concise. I think the main problem is that there is an extraordinary amount of detail in the main text regarding the various parameters used for modeling, rather than focusing on the results of the modeling. In this regard, the manuscript would be easier to read if the authors could please move modeling methods-related material either to the methods section or to a supplementary methods section."*

We took these comments into account and tried to simplify the Results section in many ways. In addition to these more specific changes, we also generally shortened and simplified various

sentences of each of the subsections of the Results, especially the transitions between paragraphs. All these minor changes are identified in the annotated PDF produced using latexdiff.

1. We simplified the description of the initialization of the simulations which is included in the section "A minimal model of post-duplication expression evolution". Instead of explaining how the randomized ancestral singleton genes were obtained, we now simply mention that simulations were initialized from randomized singletons which were duplicated into two paralogs retaining the ancestral transcription and translation levels, while referring to the Methods for further information. Lines 249-250 now read: "*Each simulation run was initialized from randomly generated singleton genes, each duplicated into two paralogs retaining the ancestral $\beta_m$ and $\beta_p$ rates (Methods).*"

2. We shortened and simplified the explanation of the mutation-selection process included in the main text. Instead of describing it step by step, we focus on the most important elements: mutations affecting either transcription or translation for one paralog are sampled randomly, following relative probabilities dictated by the relative mutational target sizes of the two traits, and are then filtered by selection. This simplified explanation comprises lines 265-278 in the new version of our manuscript. In addition, we extended the caption of Fig 2C, such that it described more precisely the mutation-selection process as illustrated by this schematic.

3. In the section "The precision-economy trade-off promotes transcriptional divergence", we removed methodological details about the "mock" simulations described and moved this information to the caption of Fig 3A, which now includes "*The standard deviation of mutational effects was set to an arbitrarily small magnitude ($\sigma_{mut} = 0.025$) and a scenario of high selection efficacy was considered ($N = 10^6$)*".

4. In the section "Selecting a biologically plausible parameter space", we removed the explanation of how the best-fitting $\sigma_{mut}$ was defined. We instead write "*To ensure the robustness of the identification of the best-fitting $\sigma_{mut}$, it was combined with this screening into a grid search, which was performed separately for the two scenarios of selection efficacy (Methods)*" (lines 363-366). We also reworked the corresponding subsection of the Methods ("Identification of the best-fitting standard deviation of mutational effects"), to ensure that it was clear and contained all the necessary details (lines 1100-1107). The caption of S6 Fig was additionally modified slightly, to more clearly explain how it shows which value of $\sigma_{mut}$ is best-fitting (see lines 1231-1235).

5. We reworked the section "*A difference of mutational target sizes may better explain the observed divergence patterns*" to make it more linear and, thus, easier to read. We now avoid explaining in-depth how simulated and empirical divergence patterns were compared before presenting the relevant comparisons. Instead, the use of the Kolmogorov-Smirnov statistics is briefly explained before the relevant figure is described (lines 384-387). Similarly for the use of the divergence correlations, their calculation is only mentioned

right before the relevant comparisons are made (lines 413-416). In accordance with these changes, we also reworked the caption of Fig 4A, so that it more precisely describes what is shown on the schematic. It now reads: "*For each combination of model and parameters, three replicate simulations of 2500 paralog pairs were performed. Two types of summary statistics were computed to compare simulation results to empirical observations. The Kolmogorov-Smirnov (KS) statistic, equal to the largest difference between two cumulative distribution functions, was used to quantify the distance between simulated and empirical distributions of log2-fold changes in transcription, translation and protein abundance (top). For each replicate simulation, the three resulting KS statistics were combined into a single mean value. The two divergence correlations between transcriptional and translational changes were also calculated on the set of paralog pairs obtained from each simulation, resulting in three measurements for each combination of model and parameter values (bottom). Created with BioRender.com*".

6. We removed superfluous methodological details from the section "*Revisiting the hypotheses when considering transcription-translation couplings and biased mutational effects distributions*". Instead of describing how the modeling of mutational target sizes had to be modified to accommodate bivariate mutational effects, we only mention that things had to be done differently in refer to the Methods for further details. We now write: *"(...) while correlations between transcriptional and translational mutations were added using a bivariate normal distribution of mutational effects. Because the latter modification meant that each mutation now affected both transcription and translation, differences of mutational target size between the two traits had to be modeled differently, using the effect size of mutations (Methods). An additional grid search was also required to identify the best-fitting standard deviations of mutational effects to use in subsequent simulations (S10_Fig)"* (lines 493-500). The caption of S10 Fig has also been extended to ensure that the "*Reference* $\sigma_{mut}$" label of the y axis is clear. We now write: "*Under this framework, standard deviations* $\sigma_{\beta m}$ *and* $\sigma_{\beta p}$ *of transcriptional and translational effects are set by the relative mutational target sizes* $P_{\beta m}$ *and* $P_{\beta p}$*, but their precise values are chosen to result in the same mean change of protein abundance per mutation as a reference* $\sigma_{mut}$ *(shown on the figure) in the univariate implementation (Methods)*" (lines 1278-1282).

Other changes:

1. We have updated the Acknowledgements section to thank the reviewers for their insightful comments which helped us greatly improve our manuscript: "*We also thank Jean-François Gout and two anonymous reviewers for their valuable comments which vastly improved this manuscript*" (lines 1370-1372)

2. We have reworked Fig 2 to ensure a uniform use of fonts throughout its panels.

3. We reorganized Fig 4 and Fig 5 so that the schematics of panels A could be bigger. Accordingly, new versions of the corresponding schematics have been made, oriented vertically rather than horizontally.

4. Minor corrections have been made to S1 Fig (to correct p-value annotations which were written as “p = ” instead of “p-val = ” like elsewhere in the paper) and S10 Fig (to remove erroneous axis labels on panels B and D). Notebooks generating S6, S13 and S15 Figs were also corrected, as y axis ticks labels were not displayed correctly upon re-execution.

5. The code deposited on Github has been updated to include these corrected notebooks. The notebook for S9 Fig has in addition been modified, to correct a mistake in a comment. Minor changes have also been made to the main script “Genome_script.py” and the “evol_funct.py” file. This was done to remove outdated code that had already been commented out before we submitted our work, as well as to ensure that automated figure generation steps at the end of the simulation script (which were not used for the simulations presented in the current paper) work as originally intended.
